# Chlamydia vaughanii sp. nov., a novel Chlamydia isolated from a tropical fish (bushymouth catfish)

**DOI:** 10.1099/ijsem.0.006753

**Published:** 2025-07-09

**Authors:** Bastian Marquis, Carole Kebbi-Beghdadi, Trestan Pillonel, Maja Ruetten, Pedro Marques-Vidal, Sebastien Aeby, Gilbert Greub

**Affiliations:** 1 Center for Research on Intracellular Bacteria (CRIB), Institute of Microbiology, Lausanne University Hospital and Lausanne University, Lausanne, Switzerland; 2 PathoVet SA, Tagelswangen, Switzerland; 3 Internal Medicine, Department of Medicine, Lausanne University Hospital and University of Lausanne, Lausanne, Switzerland

**Keywords:** *Chlamydia*, *Chlamydiales*, evolution, fish, intracellular bacteria, vertebrates

## Abstract

The *Chlamydiaceae* is a family of strict, intracellular bacteria which include human and animal pathogens such as *Chlamydia trachomatis* and *Chlamydia psittaci*. Following the death of multiple *Ancistrus dolichopterus* fish (bushymouth catfish) in a tropical aquarium, the specimens were examined for a potential infectious agent. To do so, McCoy cells (ATCC CRL-1696) were inoculated with samples isolated from the specimens and became infected by an intracellular bacterium. The entire genome of the infectious agent was sequenced (study accession PRJEB69484) and, based on nine taxonomic markers, was classified as a novel species belonging to the *Chlamydia* genus (DSMZ no. 117479, CSUR no. QA1836). We propose the name *Chlamydia vaughanii* sp. nov., in memory of the late Professor Lloyd Vaughan. *C. vaughanii* has the largest genome (1.3 Mbp) of the *Chlamydia* genus. This appears to be a consequence of multiple duplications in genes encoding putative adhesins. Like other pathogenic *Chlamydia*, it can infect mammalian cells, but it cannot infect either insect or amoeba cells. It additionally can grow in Epithelioma papulosum cyprinis (EPC) cells (fathead minnow, ATCC CRL-2872) but only when cultivated at 30 °C. We developed a *C. vaughanii*-specific quantitative PCR which amplifies the *mutS* gene and analysed several samples from the aquarium. * C. vaughanii* was retrieved from all deceased *A. dolichopterus* fish, but not from any other sample in the aquarium, suggesting that it indeed originated from the fish and was not a contaminant. *C. vaughanii* is the first *Chlamydia* isolated from fish.

## Data Summary

The supplementary data are available in FigShare https://doi.org/10.6084/m9.figshare.28424588.v [1].

## Introduction

The *Chlamydiaceae* is a family of the *Chlamydiae* phylum notable for containing common human and animal pathogens such as *Chlamydia trachomatis*, *Chlamydia abortus* and *Chlamydia pneumoniae* [2]. All bacteria from this phylum are obligate intracellular and share the same biphasic developmental cycle, with an extracellular and infectious stage, the elementary body, and an intracellular and replicative stage, the reticulate body. The different families of chlamydiae do, however, differ in terms of host range [3], with evidence suggesting that families such as the *Parachlamydiaceae* and *Criblamydiaceae* are protist symbionts [4,6] that cannot replicate in fish, mammalian or insect cell lines [7,10], while members of the *Rhabdochlamydiaceae* appear to be arthropod pathogens [11,14] that cannot multiply in amoebae [15]. On the other hand, the natural host of other families, such as the *Waddliaceae*, still remains unknown, although the ability of its members to grow in a wide variety of cell lines [10,16, 17] suggests a broad host range and a possible mammalian reservoir [18,20]. The members of the *Chlamydiaceae* could be detected and isolated from birds, reptiles [21,22] and mammals [23] but are unable to grow in amoebae [6], unlike other chlamydiae. Importantly, *Chlamydiaceae* retain the ability to jump between vertebrate species [24] and incidentally cause zoonotic diseases, such as *Chlamydia psittaci* pneumonia in humans [25]. Altogether, it appears that *Chlamydiaceae* adapted to vertebrate hosts at large, although they could never be isolated from fish despite evidence that they can infect those animals [26,27].

Chlamydiae are, indeed, known to cause disease in various fish species, notably epitheliocystis, a disease characterized by the presence of large intracellular cysts in the gill cells [28]. This disease affects both wild and farmed animals [29], and while its impact is not fully elucidated yet, epitheliocystis has been associated with peaks of mortality in aquaculture farms [28,30]. Several families of the *Chlamydiota* phylum have been associated with epitheliocystis: the *Chlamydiaceae* [31], whose member *Candidatus Clavichlamydia salmonicola* was recently sequenced [27,32, 33], the *Parilichlamydiaceae*, a deep-branching family in the *Chlamydiae* phylum that could be detected in both freshwater and marine fish, and the *Simkaniaceae*, whose member *Candidatus Syngnamydia salmonis* could be isolated from infected Atlantic salmon in an amoebal co-culture system [34,35]. Unfortunately, neither the strain nor its genome was deposited in public biobanks, preventing further studies. Although no other agent of epitheliocystis could be cultivated, metagenomic approaches allowed the sequencing of *Ca*. *Cl. salmonicola* [27] and of several species of *Parilichlamydiaceae* [36,37]. Similarly to the members of the *Chlamydia* genus, those species have a reduced genome with a limited metabolic capacity, suggesting an adaptation and likely a restriction of the host range to fish. One notable exception is *Ca*. *S. salmonis*, which reportedly harbours a 2.1 Mbp genome [34], similarly to the 2.5 Mbp genome of the closely related species *Simkania negevensis* [38]. Interestingly, both species appear to maintain a wide host range, as they are able to grow both in vertebrate cells and in amoebae [34,39]. Despite the high prevalence of epitheliocystis in aquaculture farms [40] and the suspected high economic impact of this disease [41], our knowledge of its physiopathology and of the causative organisms is limited and currently lacks *in vitro* experimental models.

In this study, we describe *Chlamydia vaughanii*, the first fish-borne species of *Chlamydia* to be isolated with a culture system. * C. vaughanii* strain CRIB76 was deposited in the DSMZ (German Collection of Microorganisms and Cell Cultures, no. 117479) and CSUR (IHU Méditerranée Infection Collection of Microorganisms, no. QA1836). This species is part of the *Chlamydia* genus and was obtained from a dead specimen of *Ancistrus dolichopterus* (bushymouth catfish), a tropical fish. It could be propagated in McCoy (ATCC CRL-1696) and EPC (ATCC CRL-2872) cells, where it displayed a peculiar inclusion morphology reminiscent of that observed in *Chlamydia caviae*. We also describe the full genome of this novel species, the largest in the *Chlamydia* genus, largely due to the duplication of genes coding for surface proteins.

## Methods

### Cell culture

Mouse fibroblast (McCoy, ATCC CRL-1696) cells were cultured at 37 °C with 5% CO_2_ in Dulbecco’s modified Eagle medium (DMEM, Invitrogen, Thermo Fisher Scientific, Waltham, MA, USA) supplemented with 10% foetal calf serum (FCS). Fall armyworm (*Spodoptera frugiperda*) ovarian epithelial cells (Sf9, ATCC CRL-1711) were maintained at 28 °C in Grace medium (Gibco, Thermo Fisher Scientific, Waltham, MA, USA) supplemented with 10% FCS. Fathead minnow (*Pimephales promelas*) cells (EPC, ATCC CRL-2872) were maintained at 25 °C in Eagle’s minimal essential medium (Invitrogen, Thermo Fisher Scientific, Waltham, MA, USA) supplemented with 10% FCS, 1% HEPES (Gibco, Thermo Fisher Scientific, Waltham, MA, USA) and 1% non-essential amino acids (Gibco, Thermo Fisher Scientific, Waltham, MA, USA). Amoebae (*Acanthamoeba castellanii*, ATCC 30010) were cultured at 25 °C in peptone-yeast extract-glucose.

### Isolation and maintenance of *C. vaughanii* (DSMZ no. 117479, CSUR no. QA1836)

The bacterium was isolated from a dead bushymouth catfish (*A. dolichopterus*) retrieved from a tropical aquarium as follows. The head of the fish was lysed in 1 ml PBS using a GentleMACS Dissociator (Miltenyi Biotec, Bergisch Gladbach, Germany). Briefly, 100 µl of the lysate was used to inoculate McCoy cells seeded the day before at 2.5×10^5^ cells well^−1^ in a 24-well plate. To prevent the growth of contaminants, the culture medium was supplemented with 25 µg ml^−1^ of streptomycin (Applichem, Darmstadt, Germany), 100 µg ml^−1^ of vancomycin (Applichem, Darmstadt, Germany), 50 µg ml^−1^ of gentamycin (Applichem, Darmstadt, Germany) and 50 µg ml^−1^ of amphotericin B (Sigma-Aldrich, Buchs, Switzerland), as recommended for the isolation of chlamydial strains [42]. Cells were centrifuged for 25 min at 2,000***g*** and then incubated for 2 h at 37 °C with 5% CO_2_. The culture medium was then replaced by fresh medium supplemented with 1 µg ml^−1^ cycloheximide (Sigma-Aldrich, Buchs, Switzerland). Five days after inoculation, cells were detached, mixed with glass beads (Sigma-Aldrich, Buchs, Switzerland) and lysed in a Precellys Evolution (Bertin Technologies, Montigny-le-Bretonneux, France) at 4,500 r.p.m. for 3×30 s with 30 s pauses between the cycles. The lysate was diluted 2× in culture medium containing antibiotics and cycloheximide (see above) and was then inoculated on fresh McCoy cells and further grown at 37 °C with 5% CO_2_. Four days after passage, bacteria could be observed under an epifluorescence microscope following staining with an anti-chlamydial HSP60 antibody. Following its isolation, *C. vaughanii* was passaged weekly as follows: infected McCoy cells were maintained at 37 °C with 5% CO_2_ before collection with a cell scraper and lysis by glass beads (Sigma-Aldrich, Buchs, Switzerland) as described above. The lysate was diluted 1:20 in DMEM supplemented with 10% FCS and 1 µg ml^−1^ cycloheximide (Sigma-Aldrich, Buchs, Switzerland) and used to infect fresh McCoy cells.

### Infection procedure

*C. vaughanii*-infected McCoy cells were collected at day 7 post-infection by scraping and lysis with glass beads as described above. The lysate was diluted to obtain an Multiplicity of infection (MOI) of 0.1–1 to infect cells seeded in 24-well plates. The MOI was estimated by infecting cells with 10-fold serial dilutions of a suspension of *C. vaughanii* elementary bodies and counting the proportion of infected cells under an epifluorescent microscope (see below). The infection was synchronized by 15 min centrifugation at 900***g***, followed by 30 min incubation at conditions normally used for the culture of the eukaryotic cell lines. To remove non-internalized bacteria, culture medium was replaced with fresh medium supplemented with 1 µg ml^−1^ cycloheximide. No cycloheximide was used for *Ac. castellanii*.

### PCR and quantitative PCR

Genomic DNA was extracted from 100 µl of the dead fish lysate following the manufacturer’s instructions (protocol for isolation of genomic DNA from animal tissues, Wizard SV Genomic DNA purification kit, Promega, Madison, WI, USA) and analysed with the 16 SigF/RP2 Chlam *Chlamydiales* PCR as described in [5].

A specific quantitative PCR (qPCR) targeting the *mutS* gene of *C. vaughanii* was developed. It was performed in a volume of 20 µl, with 10 µl of iTaq Universal Probe Supermix (Bio-Rad, Cressier, Switzerland), 5 µl of template DNA, 200 nmol l^−1^ of primers (mutS-F 5′-CCCATGGGAGGAAGATTATTACGTC-3′, mutS-R 5′-GACGTTCAAGATCACGTACCTGAG-3′) and 100 nmol l^−1^ of probe (mutS-P 5′-TCGCTGCGTCAGGATGCTGT-3′). The cycling conditions consisted of 5 min of initial denaturation at 95 °C, followed by 40 cycles of 15 s denaturation at 95 °C and a 1 min hybridization and elongation step at 60 °C. The pan-*Chlamydiales* qPCR was performed as previously described [43]. All qPCRs were performed on a QuantStudio3 real-time PCR system (Applied Biosystems, Thermo Fisher Scientific, Waltham, USA).

### Growth kinetics

Cells (Sf9, EPC, McCoy and *Ac. castellanii*) were seeded in 24-well plates at a density of 10^5^ cells per well and incubated at their usual growth temperature before infection with *C. vaughanii* at an MOI of 0.1–1, following the procedure described above. Samples were taken at 0, 6, 30, 54 and 78 h to quantify the growth, either with the *C. vaughanii mutS* qPCR or by immunofluorescence. For qPCR, the cells were detached with a cell scraper. The DNA was extracted from 100 µl of cell suspension manually using the Wizard SV Genomic DNA Purification Kit (Promega, Duebendorf, Switzerland) or on a QIAcube automated system using the QIAamp DNA Mini QIAcube Kit (Qiagen, Hilden, Germany) following the manufacturer’s protocol for bacterial pellet DNA extraction. The doubling time was estimated by dividing 24 h by the largest log_2_ difference of genome copies between consecutive time points.

### Immunofluorescence

Cells (Sf9, EPC, McCoy and *Ac. castellanii*) were plated on glass coverslips in 24-well plates at a density of 2×10^5^ cells per well. They were infected with *C. vaughanii* at an MOI of 0.1–1 as described above. The infected cells were fixed at various time points post-infection with ice-cold methanol for 5 min before being washed 3× in PBS. They were then blocked in PBS+0.1% saponin+10% FCS+0.01 NaN_3_ (blocking solution) for at least 2 h, before a 2 h incubation with the primary antibody (in-house anti-*Si. negevensis* HSP60) diluted at 1:200 in blocking solution. After three washes in PBS+0.1% saponin, coverslips were incubated for 1 h at room temperature in blocking solution with 1.6 µg ml^−1^ 4',6-diamidino-2-phenylindole (DAPI) dilactate (Molecular Probes, Thermo Fisher Scientific, Waltham, USA), 100 µg ml^−1^ Texas red conjugated-Concanavalin A (Invitrogen, Thermo Fisher Scientific, Waltham, USA) and Alexa-488 conjugated goat anti-mouse antibodies diluted 1:1,000 in blocking solution (Life Technologies, Thermo Fisher Scientific, Waltham, USA). Coverslips were finally washed 2× in PBS and 1× with water, embedded in Moewiol (Sigma-Aldrich, Buchs, Switzerland) and kept at 4 °C in the dark. The coverslips were imaged with a Zeiss LSM 900 confocal microscope (Zeiss, Feldbach, Switzerland).

### Electron microscopy

McCoy cells were seeded in T25 flasks at a density of 4×10^5^ cells ml^−1^ and infected with *C. vaughanii* at an MOI of 0.1–1. The cells were collected by scraping the wells at various time points and pelleted by a 10 min centrifugation at 500***g*** before resuspension in 0.1 mol l^−1^ phosphate buffer (PB) at pH 7.4 with 4% paraformaldehyde (Electron Microscopy Science, Hatfield, PA, USA) and 2.5% glutaraldehyde (Fluka, Buchs, Switzerland). The cell suspension was incubated at 4 °C for 4 h. Infected cells were then washed in PB and re-suspended in PB before being sent for preparation at the Lausanne University Electron Microscopy facility. The samples were examined with a Philips CM100 1201 transmission microscope (Philips, Eindhoven, The Netherlands).

### Sequencing

Two T75 flasks of infected McCoy cells were collected by scraping at day 7 post-infection. Cells were lysed with glass beads as described above. DNA was extracted from the lysate using a QIAamp DNA Mini Kit (Qiagen, Hilden, Germany) on a QIAcube robot following the manufacturer’s protocol for bacterial pellet DNA extraction. The sample was then depleted from host cell methylated DNA using the NEB Next Microbiome Kit (New England Biolabs, Ipswich, MA, USA) following the manufacturer’s protocol before undergoing a final purification step using CleanNGS magnetic beads (CleanNA, Waddinxveen, The Netherlands). DNA was then quantified with a Qubit dsDNA Quantification Assay Kit (Invitrogen, Thermo Fisher Scientific, Waltham, USA). Genomic libraries were prepared using the Nextera XT Kit (Illumina, San Diego, CA, USA) and sequenced on a MiSeq platform (Illumina, San Diego, CA, USA) to produce 250 bp paired-end reads.

### Assembly

The reads were trimmed with fastp [44] (v0.23.2) with default parameters. The trimmed reads were then assembled with SPAdes [45] (v3.14 with option – isolate). The resulting assembly graph was visualized with Bandage [46] (v0.8.1). blastn [47] (v2.13.0) searches were used to filter out contigs with homology to the mouse genome (GCF_000001635.27). Two PCR assays were then used to fill the contig gaps in the assembly. The primers are listed in Table S1, available in the online Supplementary Material. The reactions were performed in a volume of 20 µl, with 12 µl of G2 Hot Start green Master Mix polymerase (Promega AG, Duebendorf, Switzerland), 1 µl of template DNA, 5.5 µl of molecular biology grade water and 250 nmol l^−1^ of forward and reverse primers (one PCR for each of the four combinations of primers). The PCR programme consisted of 2 min at 95 °C followed by 35 cycles of 30 s hybridization at 60 °C, 2 min elongation at 72 °C and 30 s denaturation at 95 °C with a final extension step of 10 min at 72 °C. The amplicons were sequenced by the Sanger method (Microsynth AG, Balgach, Switzerland). The contigs were manually assembled in Geneious based on the sequences obtained from the sequencing PCR. To determine the depth of sequencing, the trimmed reads were mapped to the assembly with BWA [48] (v0.7.17) and the mapping was examined using QualiMap [49] (v2.2.2). The synteny to closely related genomes was assessed with Mauve [50] (v2.4.0). The assembly was annotated with PGAP (build 5508) [51].

### Taxonomic classification

The taxonomic classification was performed using nine marker genes validated for the taxonomy of the *Chlamydiales*, according to Pillonel *et al.* [52]. The markers were identified with hmmsearch (v3.3) using previously published models [52] and compared to their homologs in RefSeq reference genomes of the *Chlamydiales* order (Table S2). The sequences were aligned with Needle (EMBOSS v6.6.0) and the alignment was used to compute the pairwise identity. The species phylogeny was inferred using IQ-TREE v2.2.0 [53], with 10,000 ultrafast bootstrap replicates [54], using a concatenated alignment of 237 single-copy orthologs identified by OrthoFinder v2.5.2 [55] aligned with MAFFT v7.49 [56]. The evolutionary models were chosen by ModelFinder [57] for each partition of the concatenated alignments. The phylogenetic tree was visualized with zDB v1.1.1 [58] and iTOL [59]. The average nucleotide identity (ANI) was calculated using PYANI (v0.2.10) with default parameters [60].

### Comparative genomics

The annotated assembly was compared to the RefSeq reference genomes of the *Chlamydiales* order (Table S2) using zDB [58]. Briefly, zDB performs an initial quality check with CheckM v1.1.3 [61] and predicts the orthology with OrthoFinder. Orthologs are then aligned with MAFFT, and phylogenetic trees are inferred from the resulting alignments with FastTree v.2.1.8 [62]. All genomes were finally annotated with COG orthologs [63], KEGG orthologs [64] and Pfam domains [65]. The COG annotation was performed with rpsblast searches using the COG models of the Conserved Domain Database (v3.20) [66], while the KEGG orthologs were assigned using Kofamscan v1.3.0 [67] and the HMM profiles of the KOfam database (version 2022-03-01). Finally, Pfam domains were assigned using the pfam_scan v1.6 helper tool and the HMM profiles of the Pfam database (release 35.0) [65]. The results were then visualized in the web interface of zDB. The orthology inference of genes predicted to be unique or missing from *C. vaughanii* was refined using tblastn and blastp searches and the comparison of synteny with homologous regions of closely related genomes.

## Results

### Isolation of a novel chlamydia from a tropical fish

Following the sudden death of 24 out of 30 bushymouth catfish (*A. dolichopterus*) from a private tropical aquarium, the animals were investigated for a causative infectious agent. Of note, no fish from the other species living in the same aquarium perished during this time (*Pterophyllum scalare*, *Hemigrammus rhodostomus* and a third undocumented species). To check whether chlamydiae could be at the origin of this epidemic, we ran a pan-*Chlamydiales*-specific PCR on DNA extracted from the lysate of a dead fish head. DNA from a member of the *Chlamydiaceae* family was found following sequencing of an amplified 1,500 bp fragment of the 16S rRNA-encoding gene, which showed over 99.5% identity with members of this family [52]. The same lysate was then inoculated onto McCoy cells and grown at 37 °C with 5% CO_2_ in the presence of an antibiotic cocktail to prevent the growth of common contaminants. In the absence of any noticeable cytopathic effects, the cells were manually lysed at day 5 post-infection, and the lysate was used to infect fresh McCoy cells. Infected cells which contained multiple inclusions filled with bacteria could then be observed 4 days later by immunofluorescence. In the absence of antibodies against this novel bacterium, we resorted to polyclonal mouse antibodies raised against the *Si. negevensis* Hsp60 protein, as such antibodies often cross-react to highly conserved antigens between different members of the *Chlamydiae* phylum [15,68]. McCoy cells were subsequently used as a culture system for this bacterium, and enough material to perform whole genome sequencing was obtained. The isolate was deposited in the German Collection of Microorganisms and Cell Culture (DMSZ) under number 117479 and in the IHU Méditerranée Infection Collection of Microorganisms (CSUR) under number QA1836.

### *C. vaughanii* is a new species of the *Chlamydia* genus

**Fig. 1.**
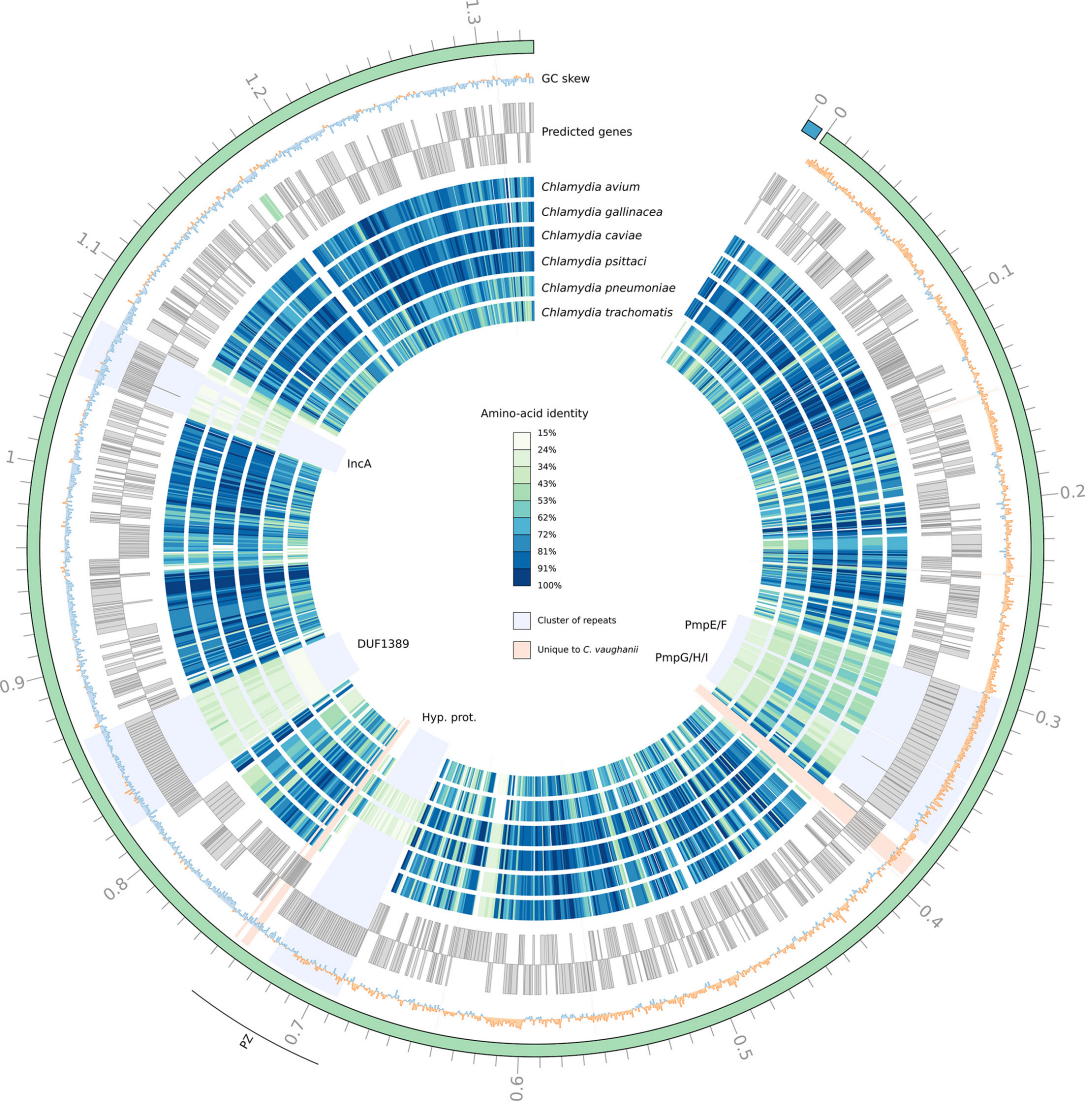
*C. vaughanii* has several clusters of repeats which are poorly conserved in closely related chlamydial genomes. The six inner circles show the amino-acid identity of the closest homologs of *C. vaughanii* in the selected genomes. The next two circles show the location of the open reading frames in both strands of the *C. vaughanii* genome. The two outermost circles show the G+C skew (orange: positive skew, blue: negative skew) and the contigs (green: chromosome, blue: plasmid). PZ: plasticity zone. Hyp. prot.: hypothetical proteins.

**Fig. 2.**
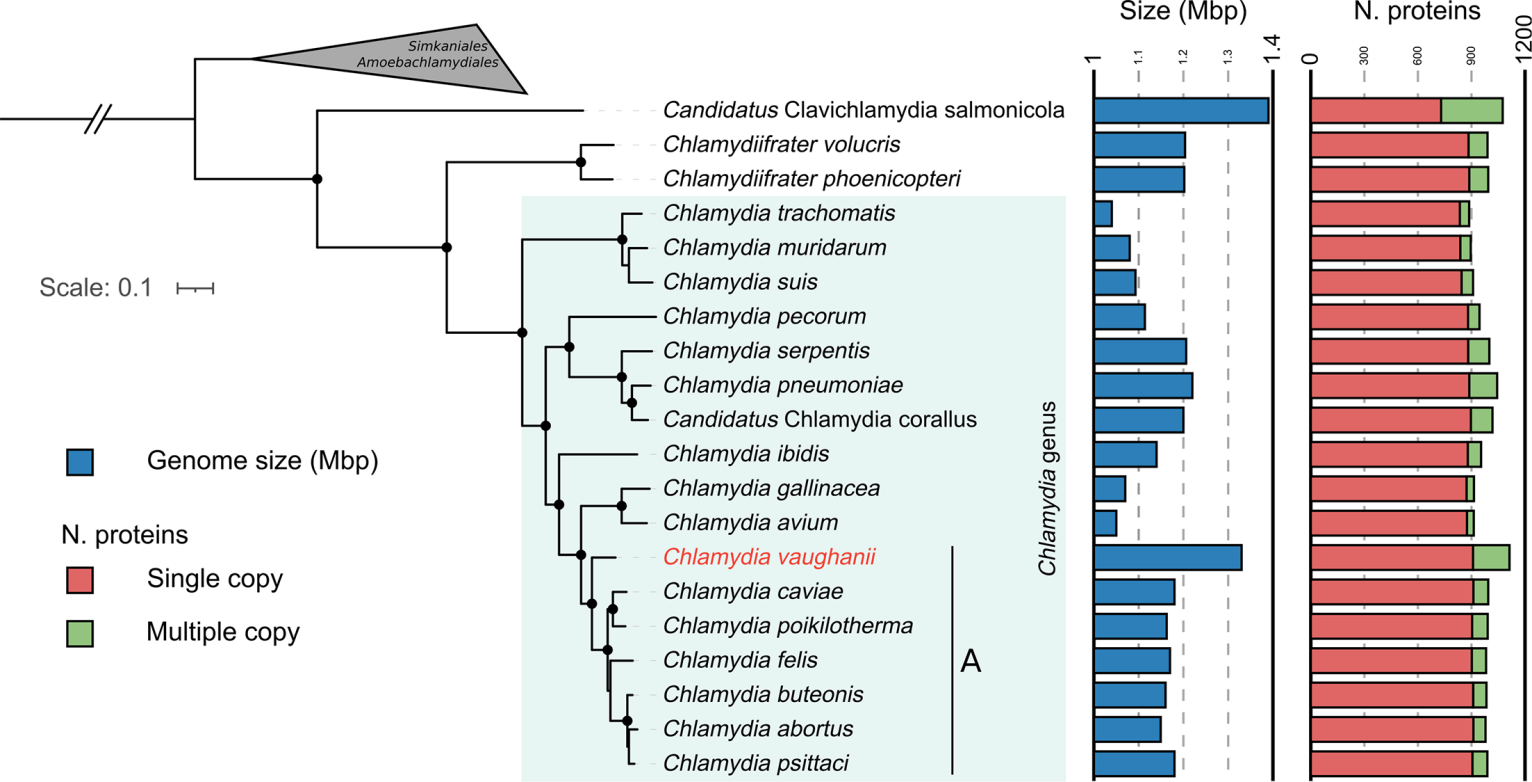
*C. vaughanii* is a new member of the *Chlamydia* genus. The figure shows a maximum likelihood phylogeny based on the concatenation of 237 single-copy orthologs. The tree is rooted on *Candidatus Similichlamydia laticola*. Black dots indicate nodes with a support higher or equal to 98% based on 10,000 ultrafast bootstrap replicates. A reduced branch length is indicated by parallel lines. The bar charts indicate the genome size and the total number of predicted proteins, distributed in single-copy proteins (red) and multiple-copy proteins (green), based on the orthology prediction of Orthofinder.

The sequencing run produced a total of 1,031,950 paired-end reads. After filtering mouse sequences and contigs smaller than 1,000 bp, we obtained two linear contigs of 986,192 and 340,182 bp and a circular contig of 7,551 bp. We could manually assemble the two linear contigs into a single circular contig of 1,326,250 bp based on the results of the sequencing PCRs (Table S1). The G+C skew (Fig. 1) and the synteny with closely related genomes (Fig. S1) confirmed the correctness of the assembly. The 7,551 bp circular contig was identified as a plasmid based on its length, higher depth of sequencing and gene content. The chromosome and plasmid have a mean sequencing depth of 241× and 496×, respectively. This isolate was classified as a new species belonging to the *Chlamydia* genus based on the comparison of nine taxonomic markers [52] (Fig. S2). The taxonomic classification was confirmed by the species phylogeny (Fig. 2). This new species was named *C. vaughanii*, in memory of the late Professor Lloyd Vaughan, who implemented a zebrafish model to study infection with *Waddlia*, a member of the *Chlamydiae* phylum [69]. The genome has a DNA G+C content of 39.3 mol% and encodes 1,113 Coding DNA sequence (CDS) and 38 tRNA. The ANI to reference genomes of the *Chlamydiaceae* family is shown in Fig. S2. The data have been deposited in the European Nucleotide Archive at EMBL-EBI under accession number PRJEB69484 (https://www.ebi.ac.uk/ena/browser/view/PRJEB69484).

### A specific qPCR confirms the presence of *C. vaughanii* in several dead fish from the same aquarium

We developed a *C. vaughanii* Taqman-based qPCR targeting the *mutS* gene and confirmed its specificity by the absence of detectable amplification in DNA from other members of the *Chlamydiales* order (*Waddlia chondrophila*, *Parachlamydia acanthamoebae*, *C. pneumoniae* and *C. trachomatis*) (data not shown). To confirm that *C. vaughanii* was not a contaminant from the aquarium, we applied the *C. vaughanii*-specific qPCR and a pan-*Chlamydiales* qPCR [43] to the lysate from three dead fish and to various samples collected from the aquarium (algae, gravel filter and smears from the windows of the tank). All fish (*n*=3) were positive by both qPCR assays, while the other samples were either negative or only positive with the pan-*Chlamydiales* qPCR (Table S3). The detected amplification with this latter qPCR is likely due to the presence of chlamydial symbionts in water-borne amoebae. To determine if *C. vaughanii* caused epitheliocystis in the deceased fish, we performed a necropsy of one of the three fish that tested positive by qPCR. While we could not observe any pathognomonic gill cysts, their absence could be due to the advanced autolysis of the specimen.

### *C. vaughanii* growth in eukaryotic cells

**Fig. 3.**
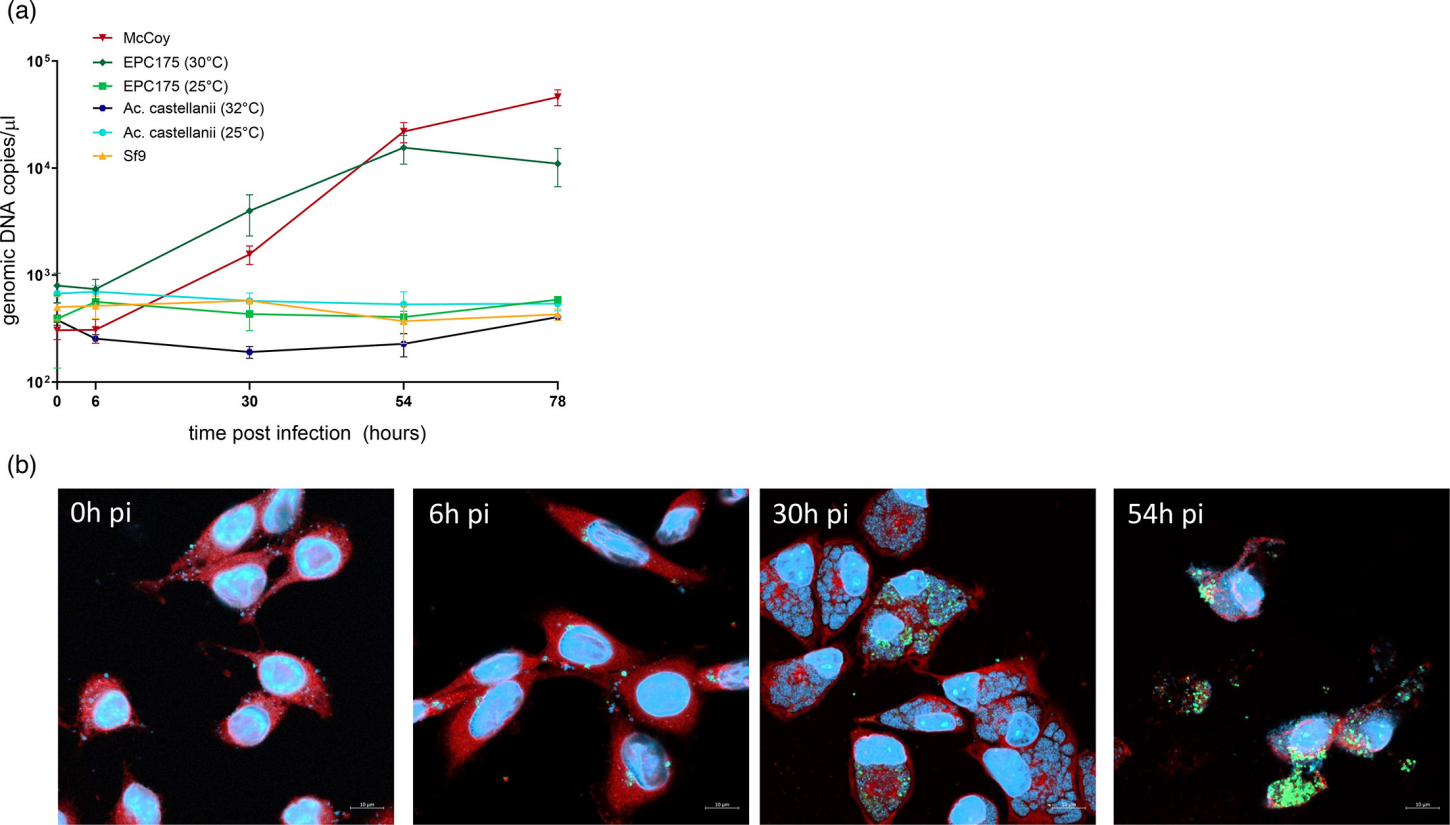
Growth kinetics of *C. vaughanii* in different cell lines. (**a**) Bacterial replication was measured by the *C. vaughanii*-specific qPCR. Results are the mean and sd of three biological replicates. (**b**) Immunofluorescence staining and confocal microscopy of McCoy cells infected with *C. vaughanii* (MOI: 0.1–1) at different time points post-infection (0, 6, 30 and 54 hpi). Bar: 10 µm. Red: concanavalin A, Blue: DAPI, Green: bacteria.

In order to better characterize the host range and infectious cycle of *C. vaughanii*, we tested the permissivity of different cell types to this novel species and assessed its growth by qPCR as well as by immunofluorescence and confocal/electron microscopy. In addition to the McCoy cells (37 °C), originally used to isolate the bacterium, permissivity was tested using a fish cell line already known to be permissive to chlamydia-related organisms (EPC, 25 °C) [10], an insect cell line (Sf9, 28 °C) and one amoebal species (*Ac. castellanii*, 25 °C). Bacterial growth could be observed in the McCoy cells with a doubling time of 6.33 h (sd=0.46 h), but not in the other cell lines (Fig. 3a). The absence of growth in EPC at 25 °C (Fig. 3a) was surprising, as this cell line was isolated from a freshwater fish and should thus offer conditions close to those found in *C. vaughanii* natural host. We hypothesized that this lack of growth could be due to the sensitivity of the bacteria to the incubation temperature, and the experiment was repeated at 30 °C instead of 25 °C. This higher incubation temperature allowed bacterial growth, with a doubling time of 9.97 h (sd=0.60 h). An increased incubation temperature, however, did not allow the growth of *C. vaughanii* in *Ac. castellanii* (32 °C) (Fig. 3a).

**Fig. 4.**
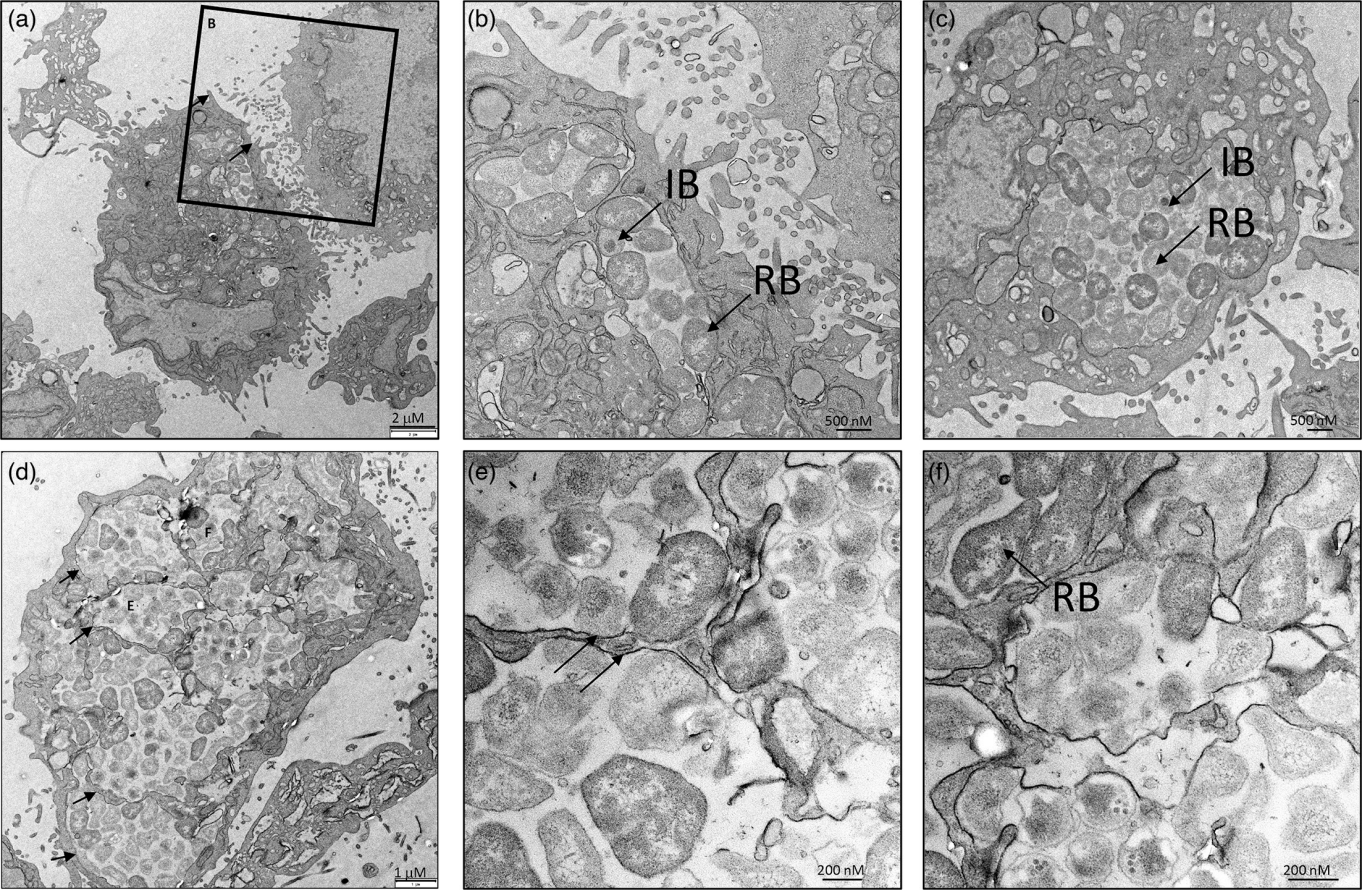
Morphology of *C. vaughanii* developmental forms and inclusions. Electron microscopy images of McCoy cells infected with *C. vaughanii* (MOI: 0.1–1) at (**a–e**) 24 h or (f) 72 h post-infection. (a) and (d) Multiple inclusions (indicated by arrows) in one cell. (b) and (c) Inclusions containing reticulate bodies (RBs) and intermediate bodies (IBs).** (e)** Two different inclusions with distinct membranes (indicated by arrows).** (f) **RBs. Bar: (a) 2 µm, (b) and (c) 500 nm, (d) 1 µm, (e) and (f) 200 nm.

The examination of infected McCoy cells by confocal microscopy revealed that *C. vaughanii* forms multiple, non-fusogenic inclusions, although cells were infected with an MOI of 0.1–1 (Fig. 3b). This also appears to be true in EPC cells (Fig. S3). This observation was confirmed by a 3D reconstruction of an infected McCoy cell that clearly showed multiple individual inclusions (Movie S1). Electron microscopy images of infected McCoy cells (Fig. 4) also confirm that multiple inclusions, surrounded by distinct membranes, are present in a single cell (panels A, D and E). The micrographs in panels B, C and F show different morphological forms of the bacteria. In particular, reticulate bodies (RBs) could be identified based on their similarity to RBs of other species of chlamydia, with a dividing RB visible in panel F. Intermediate bodies can also be observed, with a distinctive central electron-dense spot. Intermediate bodies are an intermediate form between the replicative RBs and the non-dividing Elementary bodies (EBs). Interestingly, *C. vaughanii* RBs do not always appear as round particles but rather display a large variety of morphologies (panel E). It is unclear whether this reflects true variations in RB morphology or if this is an artefact of specimen preparation [70]. We tried to visualize EBs purified from culture medium but were unable to obtain a good-quality micrograph.

### *C. vaughanii* has the largest genome of the *Chlamydia* genus

As *C. vaughanii* is the only member of the *Chlamydia* genus isolated from a fish, we compared its genome to the genomes of other members of the *Chlamydiales* order (Table S2) to identify potential genomic determinants of its host range. The first difference is that *C. vaughanii* has a larger genome than the other members of the *Chlamydia* genus (Fig. 2). This appears to be the consequence of multiple gene duplications. Indeed, the size of the single-copy proteome is similar to that of the other members of the genus, while multiple-copy proteins account for most of the variation in terms of the number of predicted proteins (Fig. 2). Moreover, only 15 orthogroups, accounting for a total of 31 proteins, were predicted to be unique to *C. vaughanii* (Table S4). Closer examination revealed that they were likely orthology inference errors, either due to gene duplications (Figs S4–S6) or to small protein size (Table S4). Conversely, only three genes were predicted to be missing from the genome of *C. vaughanii*. The absence of those three genes from *C. vaughanii* is likely related to the same orthology inference errors reported above, as they are located in regions whose homologs in *C. vaughanii* contain orthologs unique to that species (Figs S4 and S6). Altogether, the gene content of *C. vaughanii* is largely similar to that of the other members of the *Chlamydia* genus, and the larger genome size of this species appears to be due to gene duplications rather than to the presence of unique genes.

The members of five orthologous groups account for more than half (121/204) of the duplicated genes in the genome of * C. vaughanii* (Fig. S7). A majority of those genes are homologs of surface proteins that are also present as multiple copies in other closely related bacterial genomes, but to a lower extent (Fig. S7). In particular, homologs of *C. psittaci* IncA account for 19 of the duplications, while homologs of different families of Pmps account for 17 (homologs of PmpG, H and I of *C. trachomatis*) and 16 (homologs of PmpF and E of *C. trachomatis*) duplications. However, the function of the two orthogroups with the highest number of duplications is unknown, with 36 copies from an orthogroup containing proteins annotated with a domain of unknown function (DUF1389) and 32 copies from another orthogroup containing unannotated hypothetical proteins encoded in the plasticity zone (Figs 1 and S8). The repeats appear in clusters in the genome of *C. vaughanii* and are poorly conserved compared to their homologs in other chlamydial genomes (Fig. 1) and among themselves (Fig. S9). In addition, pseudogenes and short proteins predicted to be unique to *C. vaughanii* are also found intertwined in the repeats, suggesting that some paralogs underwent degradation after their duplication (Figs S8 and S10–S12). Similar repeats are also present in homologous regions of closely related genomes (Figs S8 and S10–S12). As suggested by the phylogeny of the genes (Figs S13–S17), the duplications appear to have arisen both before and after speciation events.

As expected, the gene content of the plasticity zone also differs from closely related species. The plasticity zone was identified as a stretch of 61 kbp between the guaA/B/adenosine deaminase and accB/C genes [21] and lacked the tryptophan operon, cytotoxin and perforin genes (Fig. S8). Those genes could not be detected anywhere else on the chromosome or the plasmid. The 7,551 bp plasmid contains 8 CDS, homologs to the well-conserved genes of the known chlamydial plasmids.

## Discussion

In this study, we report *C. vaughanii*, the first chlamydia to be isolated from a fish. Unlike the other known fish-borne chlamydiae [27,36], this bacterium belongs to the *Chlamydia* genus. As other members of this genus were isolated from mammals, birds, amphibians [71] and reptiles [22], our finding suggests that the host range of *Chlamydia* includes organisms in the whole Vertebrata phylum. Surprisingly, the genome of *C. vaughanii* is the largest described to date in the *Chlamydiaceae* family due to the expansion of several gene families, mostly homologs of known surface proteins. Otherwise, the gene content is similar to that of the other species of the *Chlamydia* genus, suggesting that the adaptation to fish is likely not due to gene loss or to the acquisition of genes through horizontal gene transfer [27].

The isolation of *C. vaughanii* from fish tissue lysate and its detection by PCR in several dead specimens suggest that this bacterium multiplies in *A. dolichopterus*. The advanced autolysis of the dead specimen, however, prevented us from confirming this finding with histopathological evidence. In particular, the possibility of *C. vaughanii* being a contaminant from another host, such as a water-borne protist, cannot be ruled out in the absence of microscopic evidence of infection. However, the absence of amplification with the *C. vaughanii* qPCR in all the other samples taken from the aquarium, together with the high number of genome copies detected in multiple fish samples, makes the possibility of contamination unlikely (Table S3). In addition, while *Chlamydiales* are known to be present in marine and freshwater environments and can be isolated from marine protists [72], the *Chlamydiaceae* appear to be fundamentally associated with animal hosts [73,75] and cannot be grown in amoebae [6], making it unlikely that *C. vaughanii* was an accidental water-borne contamination. In addition, we also demonstrated that *C. vaughanii* is unable to multiply in *Ac. castellanii* at 25 °C and at 32 °C. Finally, the growth of this bacterium in fish and mammalian cell lines also suggests its ability to inhibit apoptosis during infection, a trait seemingly necessary for chlamydiae to be able to multiply in multicellular organisms [7,8], which is absent in amoebal symbionts. Although *C. vaughanii* could not be directly observed in the tissues of dead *A. dolichopterus*, the inability of this bacterium to grow in *Ac. castellanii*, the qPCR results and the known host range of all other *Chlamydiaceae* make it likely that this bacterium indeed originated from the fish. Interestingly, the growth of *C. vaughanii* in a fish cell line appears to be temperature sensitive. This confirms previous observations that chlamydiae adapt to the temperature of their host, at the cost of the ability to multiply in organisms with a different body temperature [9,76, 77]. It is unlikely that the bacteria could propagate due to an effect of the temperature shift on the host cell itself, as EPC can be grown in temperatures ranging from 20 to 30** **°C.

The role of *C. vaughanii* in *A. dolichopterus* rapid death is less clear. While chlamydiae have been associated with epitheliocystis, this disease usually does not cause such massive mortality, except in juveniles [28,41]. The absence of gill cysts in our samples further suggests that *C. vaughanii* might not be an agent of epitheliocystis but may cause another type of disease, similarly to *Candidatus Renichlamydia lutjani* [78]. The latter was observed in the kidneys of blue-striped snappers, although it is unclear whether bacteria were also present in the gills or not [78]. It must, however, be noted that the advanced autolysis in our specimen may have prevented the detection of cysts, even if they were present. Altogether, the clinical course and the lack of microscopic evidence of epitheliocystis suggest that *C. vaughanii* might cause a yet unknown disease. Interestingly, only fish of the species *A. dolichopterus* were affected; it is therefore tempting to speculate that, much like the agents of epitheliocystis [37], *C. vaughanii* has a tropism for specific fish species. Without healthy controls, it is impossible to ascertain the pathogenic role of the bacteria, as it could cause asymptomatic infections, or its presence in dead fish could be a coincidence. Resolving this question may necessitate the use of a fish model of infection, which would also allow the determination of the natural history of the disease and the assessment of whether *C. vaughanii* is indeed an agent of epitheliocystis.

The fragmented morphology of *C. vaughanii* inclusions is also surprising and is reminiscent of the inclusions of *incA* knockout strains of *C. trachomatis* [79,80] and of the inclusions of more closely related chlamydial species, such as *C. abortus* [81] and *C. caviae* Guinea Pig Inclusion Conjunctivitis (GPIC) [82]. Similarly to what was observed for those two chlamydial species, the fragmented inclusions were consistently observed in all infected cells, despite MOIs ranging from 0.1 to 1. Such an MOI makes it unlikely that all cells harbouring fragmented inclusions were independently infected by multiple EBs. Unlike what was observed for non-fusogenic isolates of *C. trachomatis* [80], the presence of multiple inclusions, therefore, does not appear to depend on the MOI. This conclusion is consistent with that of Rockey *et al.*, who postulated that *C. caviae* inclusions separate into different ‘lobes’ during the infection cycle [82]. This hypothesis might also be true for *C. vaughanii*. The presence of clusters of bacteria in early time points (Fig. 3) made us speculate that the multiple inclusions, observed independently of the MOI, may be a consequence of the internalization of clusters of EBs that failed to separate into individual infectious particles after their host cell lysis. According to this hypothesis, EBs would develop separately in their own inclusions, similarly to non-fusogenic strains of *C. trachomatis* at high MOIs [80,82]. However, this inclusion morphology could not be observed in fish infected with either distantly related epitheliocystis agents [83,85] or with the more closely related *Ca. Cl. salmonicola* [31]. It could therefore be specific to some of the species of clade A (Fig. 2). Finally, it is also possible that the imaging techniques we used failed to highlight a tubular network linking together the small inclusions into a single large multi-lobular inclusion similar to that of *Si. negevensis* [86].

The genome of *C. vaughanii* is the largest in the *Chlamydiaceae* family, largely due to gene duplications. The gene content of this new species is similar to that of closely related zoonotic species of chlamydiae which infect livestock and birds, such as *C. psittaci* [87]. In particular, we did not identify any genes unique to *C. vaughanii*, in line with previous publications that also failed to identify genes specific to fish-borne chlamydiae [27]. *C. vaughanii* does not appear to have lost any genes which are conserved in the other members of clade A (Fig. 2), suggesting that the loss of metabolic pathways observed in the *Parilichlamydiaceae* and *Cl. salmonicola* [27,37] is not a genomic hallmark of fish-borne chlamydiae. It may, however, be a consequence of a restriction in the host range of those species [27,88]. Conversely, the multiplication of Pmp genes observed in *C. vaughanii* can also be seen in *C. abortus* [89], *C. pneumoniae* [90] and *C. psittaci* [91,92] and appears to be a hallmark of chlamydiae with a large host range. Pmps are membrane proteins that play the role of adhesins [93] and have been suggested to be a determinant of tissue and host tropism [94,95]. Interestingly, the expansion of the Pmp genes is limited to the PmpG/I family in most chlamydial genomes, with little variation in the PmpE family [92], while both families are expanded in *C. vaughanii* (Fig. S7). Despite being poorly conserved, with some of its members harbouring frameshift mutations, all Pmp-encoding genes of *C. pneumoniae* were shown to be transcribed, and the majority of the proteins were expressed during infection [96]. As the Pmps of *C. vaughanii* have a similar conservation profile (Fig. S9), it is likely that the members of both the PmpG/I and PmpE/G/F families are similarly expressed in *C. vaughanii*. The presence of pseudogenes and low conservation of the amino-acid sequences in both Pmp families suggests that those proteins are under relaxed selection and likely fit a previously described evolutionary scenario of gene duplications followed by pseudogenization [97].

Massive duplications could also be observed for DUF1389-containing proteins and IncA-family proteins (Fig. S7). The DUF1389 domain was identified in Inc proteins and is unique to the *Chlamydiales* order [98], while IncA has been shown to be necessary for inclusion fusion [79,80]. Interestingly, multiple copies of IncA-family proteins appear in other chlamydiae that also display fragmented inclusions (Fig. S7), suggesting a possible association. More generally, Inc proteins are type III secretion system effectors that are inserted into the inclusion membrane after their secretion and are important mediators of host–pathogen interactions (reviewed in [99]). A similar expansion of effector proteins has already been described in other *Chlamydiae*, and it has been theorized to be a consequence of a recent shift in ecological niche [100]. The duplications of DUF1389-containing proteins, Pmps and IncA-family proteins might therefore be due to a recent diversification or change of host.

*C. vaughanii* is the first fish-borne member of the *Chlamydia* genus to be isolated in culture and sequenced. While its genome is remarkably similar to that of other *Chlamydiaceae*, the expansion of membrane and effector proteins suggests a recent host shift. Moreover, an animal model of infection will be necessary to determine the relevance of *C. vaughanii* as a fish pathogen, as well as its tissue distribution. After the recent description of chlamydiae in birds, reptiles and amphibians, the report of a fish-borne chlamydia suggests that the *Chlamydiaceae* likely co-evolved with the common ancestor of vertebrates.

## Description of *Chlamydia vaughanii* sp. nov.

*Chlamydia vaughanii* (vaug.han'i.i. N.L. gen. n. *vaughanii*, named after late Professor Lloyd Vaughan, who studied fish pathogens and developed a zebra fish model for *W. chondrophila* infections).

*C. vaughanii* was isolated from *A. dolichopterus* (bushymouth catfish) and reared in a private tropical aquarium in Switzerland. The type strain is an intracellular bacterium that could be grown in mouse (McCoy, ATCC CRL-1696) and fathead minnow (EPC, ATCC CRL-2872) cells incubated at 37 and 30 °C, respectively. *C. vaughanii* did not grow at temperatures below 30 °C. The bacteria multiply in non-fusogenic inclusions. RBs are spherical and 500–1,000 nm wide, similarly to other *Chlamydiae*. The genome of strain CRIB76 has a size of 1.3 Mbp and a 7.6 Kbp plasmid, with a G+C content of 39.3 mol%. It encodes a total of 1,113 predicted CDS and 38 tRNA. The genome sequence accession number is GCA_964023145.1. The 16S sequence is available under accession OZ026853 and the reads under accession number ERR12780012. *C. vaughanii* belongs to the *Chlamydia* genus based on the phylogenetic tree and on nine taxogenomic markers. Its closest neighbours are the members of the clade containing *C. psittaci*, *C. caviae*, *Chlamydia poikilotherma*, *Chlamydia felis*, *Chlamydia buteonis* and *C. abortus*, with a mean ANI of 82.9% to the members of this clade (range 82.7–83.3%). *C. vaughanii* strain CRIB76 was deposited in the DSMZ (no. 117479) and CSUR (no. QA1836) strain collections.

## Supplementary material

10.1099/ijsem.0.006753 Uncited Supplementary Material 1.

10.1099/ijsem.0.006753 Uncited Supplementary Material 2.

10.1099/ijsem.0.006753 Uncited Supplementary Material 3.

## Abbreviations

ANI: average nucleotide identity
DMEM: Dulbecco’s modified Eagle medium
FCS: foetal calf serum
PB: phosphate buffer
qPCR: quantitative PCR
RBs: reticulate bodies
