# Supplementary material for: Chlamydia vaughanii sp. nov., a novel Chlamydia isolated from a tropical fish (bushymouth catfish)
Source: Int J Syst Evol Microbiol. 2025 Jul 9;75(7):006753. doi: 10.1099/ijsem.0.006753 (PMC12282049; doi:10.1099/ijsem.0.006753)
Supplement: Uncited Supplementary Material 1. [file ijsem-75-06753-s001.pdf]

Supplementary materials

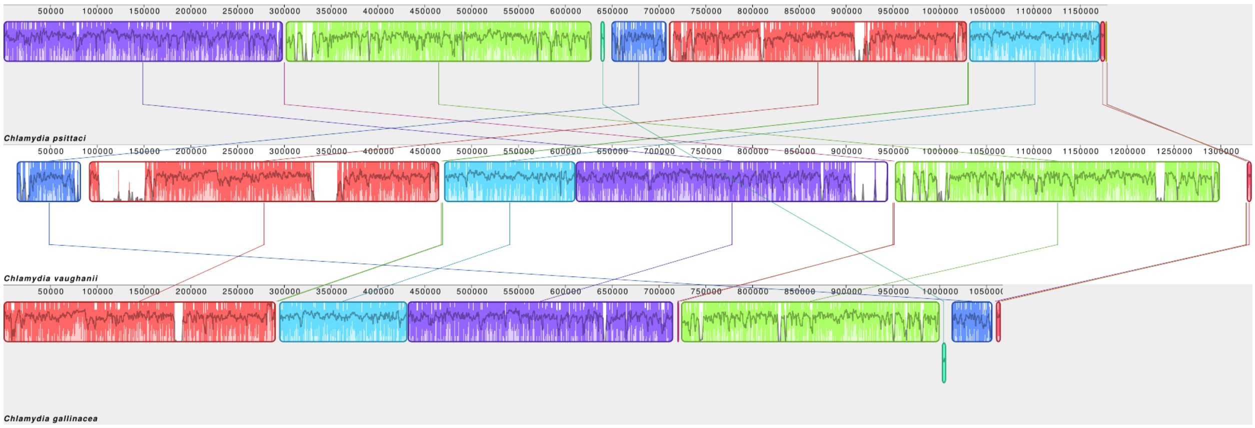

Supp. Fig. 1
Whole genome alignments of *C. psittaci* (top), *C. vaughanii* (middle) and *C. gallinaceae* (bottom), highlighting the high level of synteny between the genome of those species. The genomes were aligned using Mauve with default parameters.

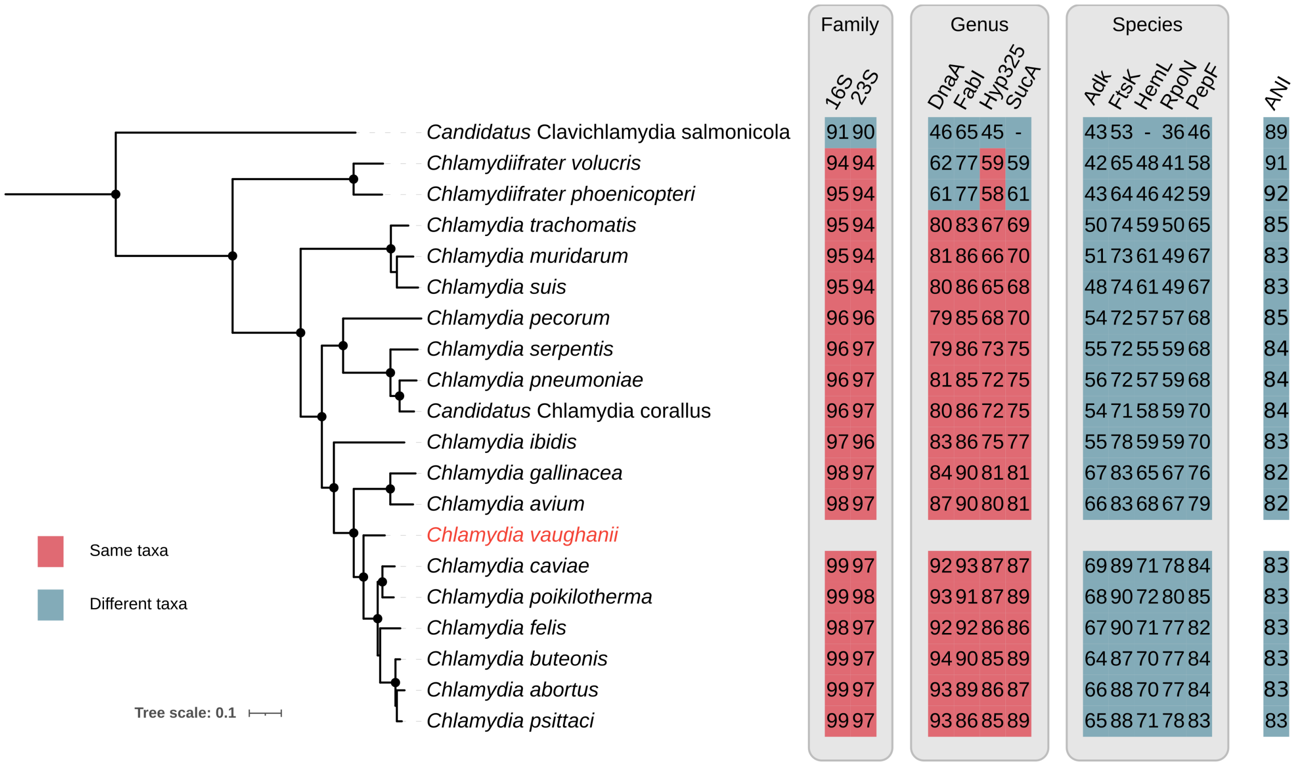

Supp. Fig. 2
Comparison of the 11 taxonomic markers developed by Pillonel et al for the taxonomic classification of chlamydia species. The plot also shows the average nucleotide identity between *C. vaughanii* and the other species. The markers were identified by the HMM models published by Pillonel et al. The pairwise identity between a marker in a given genome and its homolog in *C. vaughanni* are shown as percentage. The markers were aligned using EMBOSS Needle. The ANI was calculated using pyani.

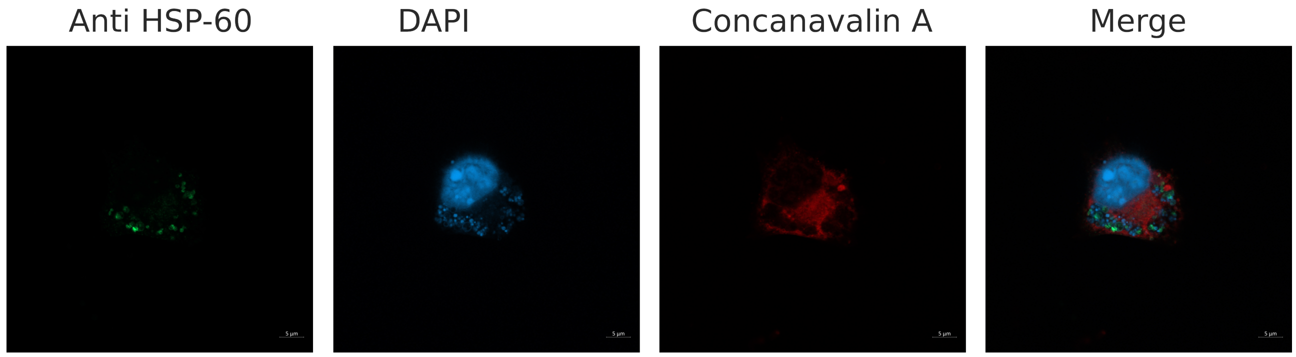

Supp. Fig. 3
Immunofluorescence staining and confocal microscopy of an EPC cell infected with *C. vaughanii* (MOI: 0.1-1) at 54 hours post-infection. Bar: 5 µm. Red: concanavalin A, Blue: DAPI, Green: bacteria.

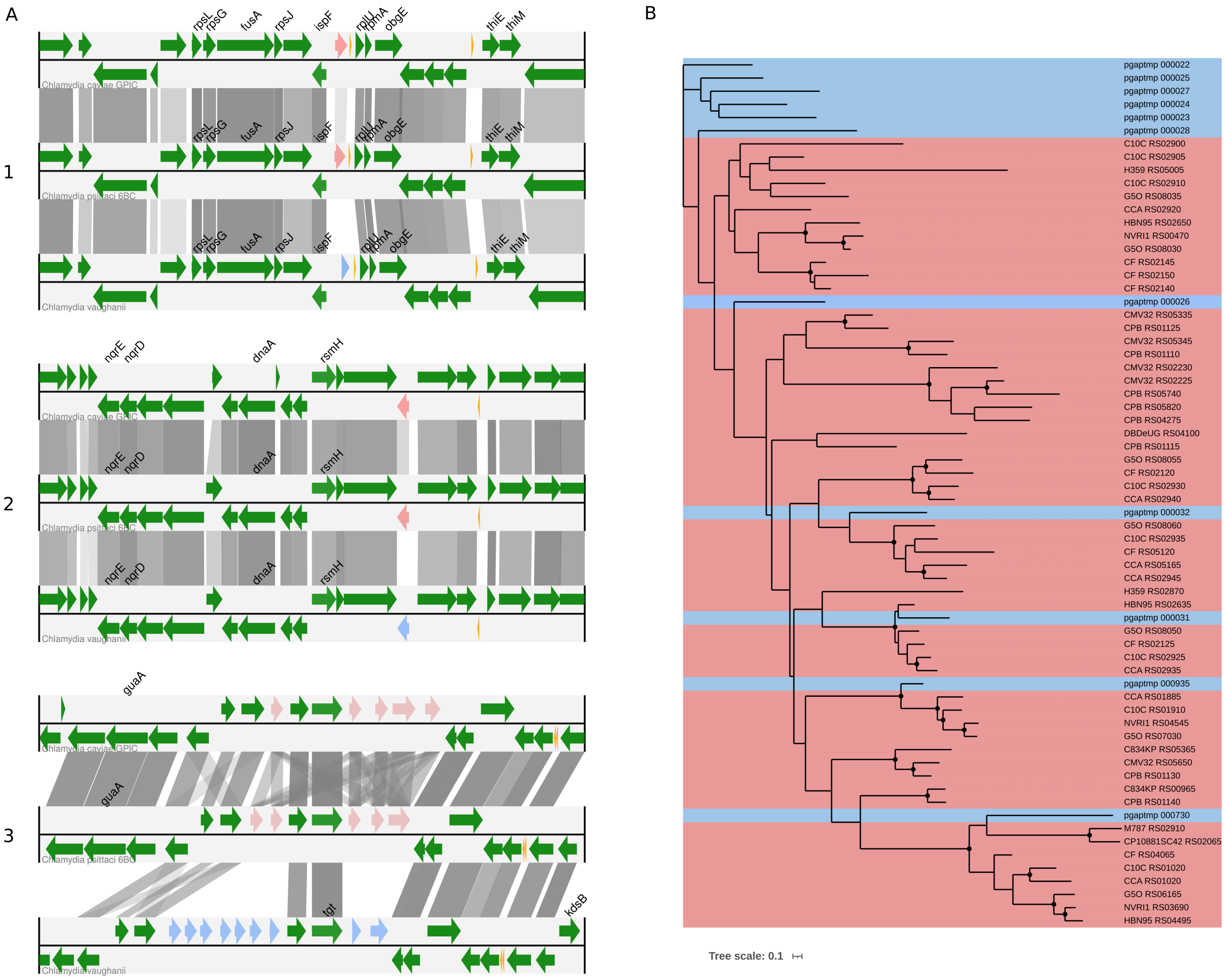

Supp. Fig. 4
A: Comparison of genomic regions of *Chlamydia vaughanii* encoding proteins of one of the orthogroup predicted to be unique to that species (blue arrows) and their homologous regions in two closely related genomes (*C. caviae*, top; *C. psittaci*, middle). Red arrow show protein coding-genes from the same orthogroup, predicted to be absent from *C. vaughanii*. Green arrows: protein encoding genes, yellow arrows: tRNA encoding genes. Orthologs in the different genomes are linked with gray bands. B: maximum-likelihood phylogenetic tree inferred from the alignment of the amino-acid sequences of the proteins from both orthogroups (red and blue arrows). Black dots

represents nodes with more than 98% support, as calculated with 1000 ultra-fast bootstrap replicates with IQ-tree. The amino-acid sequences were aligned with mafft. In blue: proteins from *C. vaughanii*. In red: proteins from other chlamydiae.

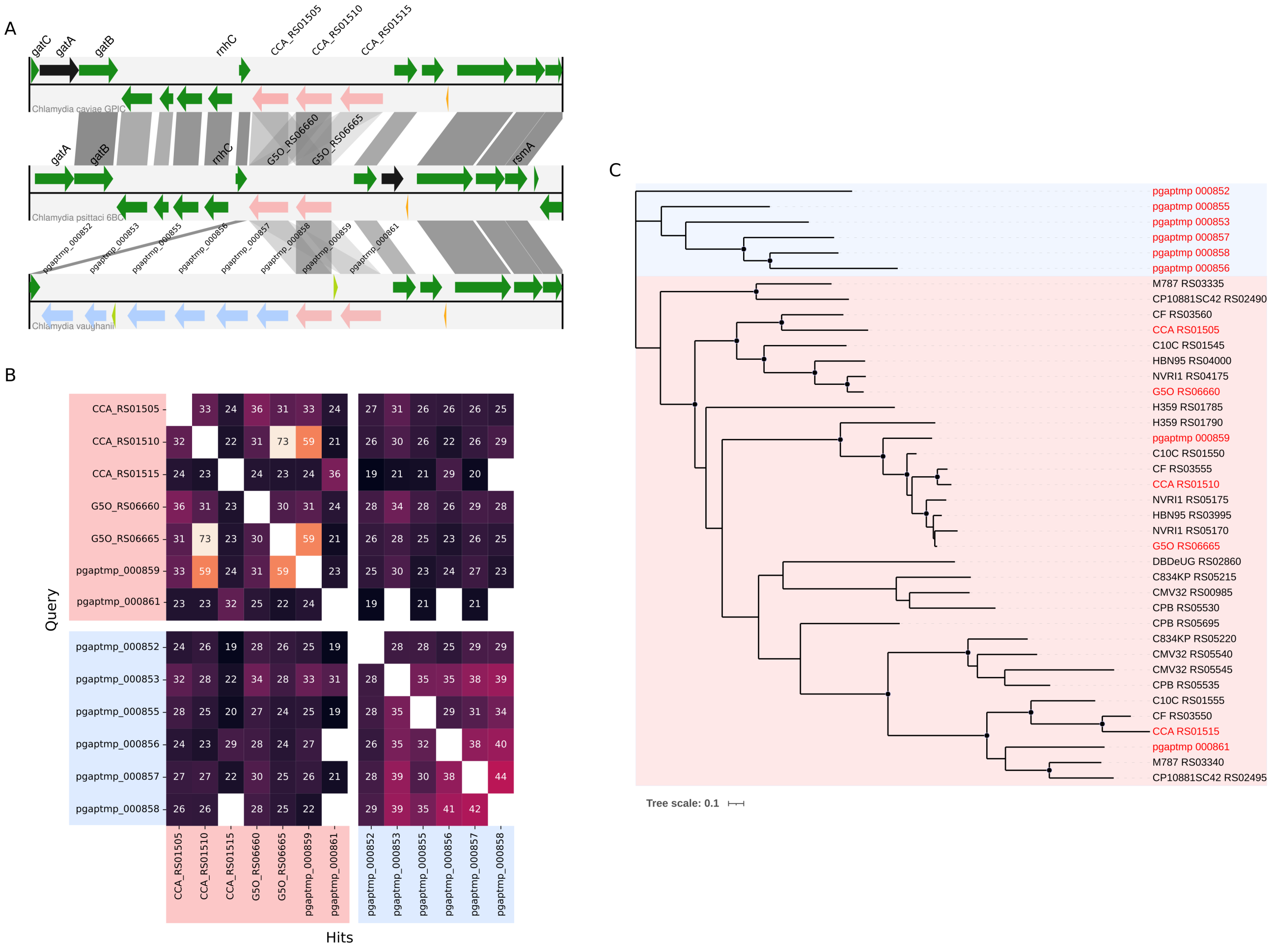

Supp. Fig. 5

Possible wrong orthology prediction due to recent duplications. A: Comparison of the genomic region of *Chlamydia vaughanii* encoding proteins of one of the orthogroup predicted to be unique to that species (blue arrows) and its homologous region in two closely related genomes (*C. caviae*, top; *C. psittaci*; middle). Dark green arrows: protein encoding genes, yellow arrows: tRNA encoding genes, black arrows: pseudogenes, light green arrow: short genes predicted to be unique to *C. vaughanii*, red arrow: protein-coding genes from the suspected true orthogroup of the genes in blue arrows. Orthologs in the different genomes are linked with gray bands. B: Heatmap of blast identities computed from an all-against-all blast search with blastp and the amino-acid sequences of both red and blue genes shown in panel A. C: maximum-likelihood phylogenetic tree inferred from the alignment of the amino-acid sequences of the proteins from both orthogroups. Black dots represents nodes with more than 98% support, as calculated with 1000 ultra-fast bootstrap replicates with IQ-tree. The amino-acid sequences were aligned with mafft. In red: the locus tags of the proteins shown in panel A and B.

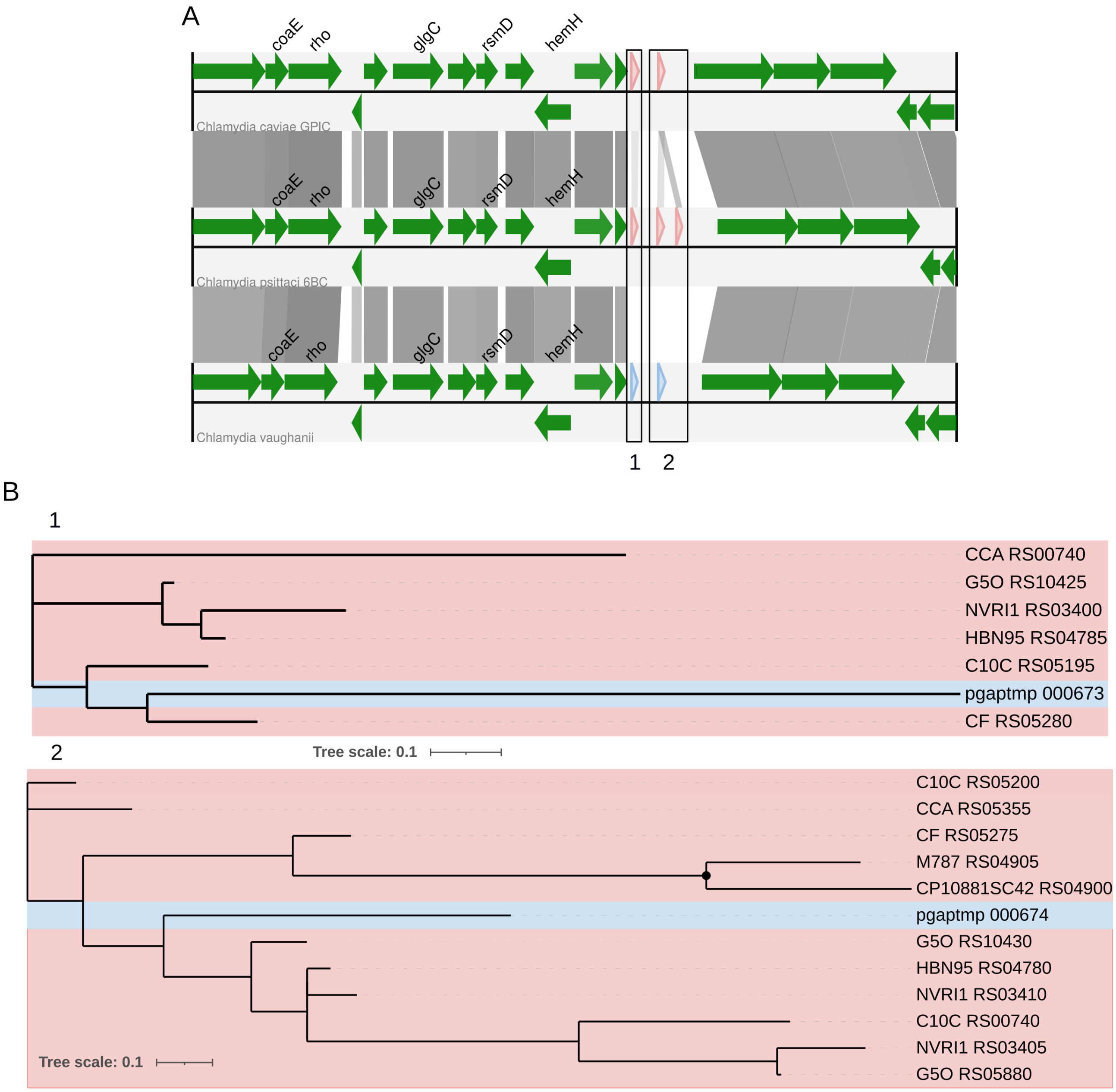

Supp. Fig. 6

A: Genomic region containing two genes encoding proteins from two different orthogroups (denoted 1 and 2) predicted to be unique to *C. vaughanii* and its homologous region in two closely related genomes (*C. caviae*, top; *C. psittaci*, middle). Blue arrow: protein-coding genes unique to *C. vaughanii*. Red arrows: protein-coding genes from the suspected true orthogroups. Green arrows: protein encoding genes. Orthologs in the different genomes are linked with gray bands. B: maximum-likelihood phylogenetic trees inferred from the alignment of the amino-acid sequences of the proteins from both orthogroups. Black dots represents nodes with more than 98% support, as calculated with 1000 ultra-fast bootstrap replicates with IQ-tree. The amino-acid sequences were aligned with mafft. In blue: proteins from *C. vaughanii*. In red: proteins from other chlamydiae.

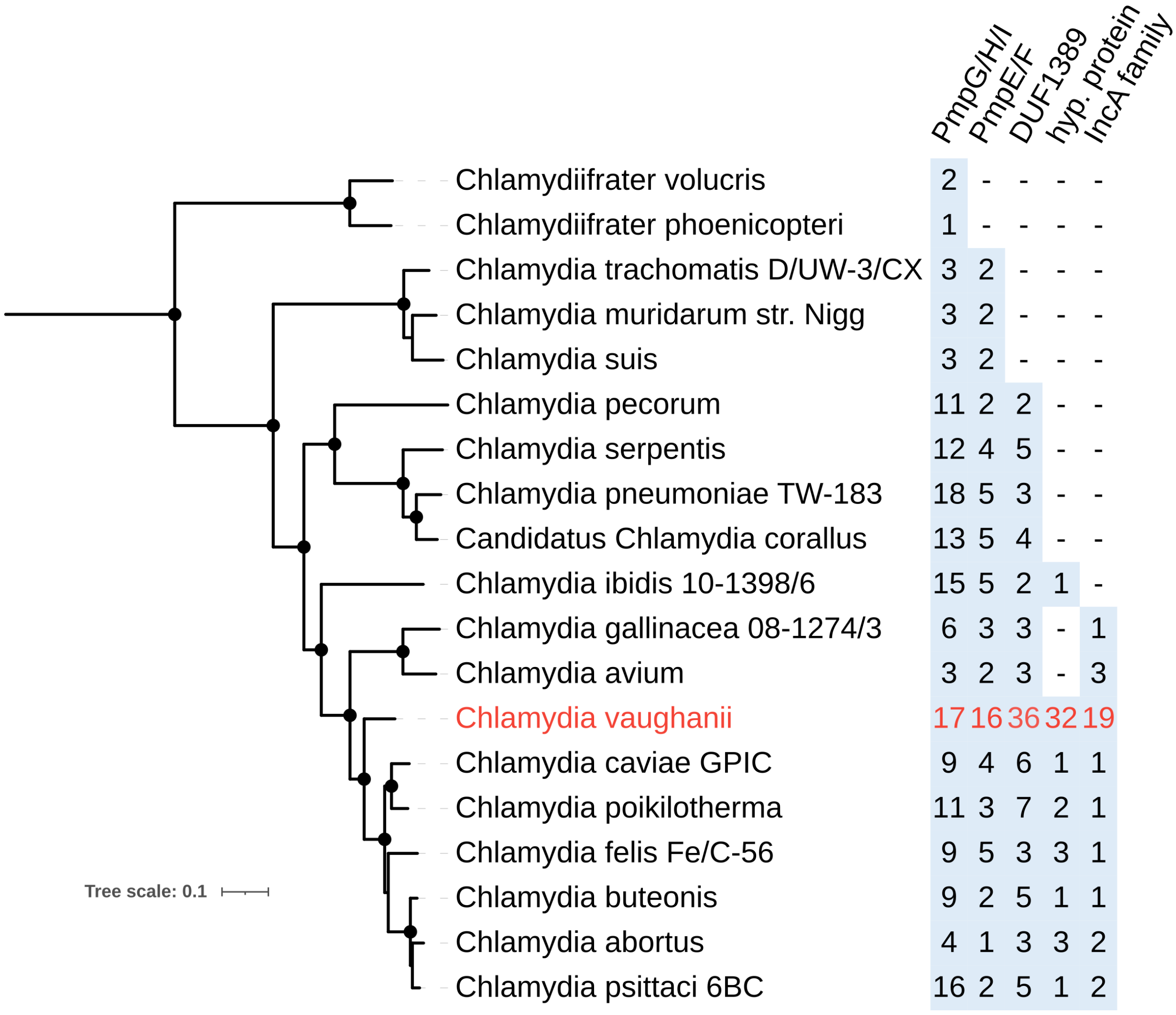

Supp. Fig. 7
Five orthogroups account for more than 50% of the repeated genes in *C. vaughanii*. The annotated tree shows the number genes in those orthogroups in the genomes of different species of the *Chlamydiaceae* family. The orthology was inferred with Orthofinder. Pmp: polymorphic membrane protein. Inc: inclusion membrane protein.

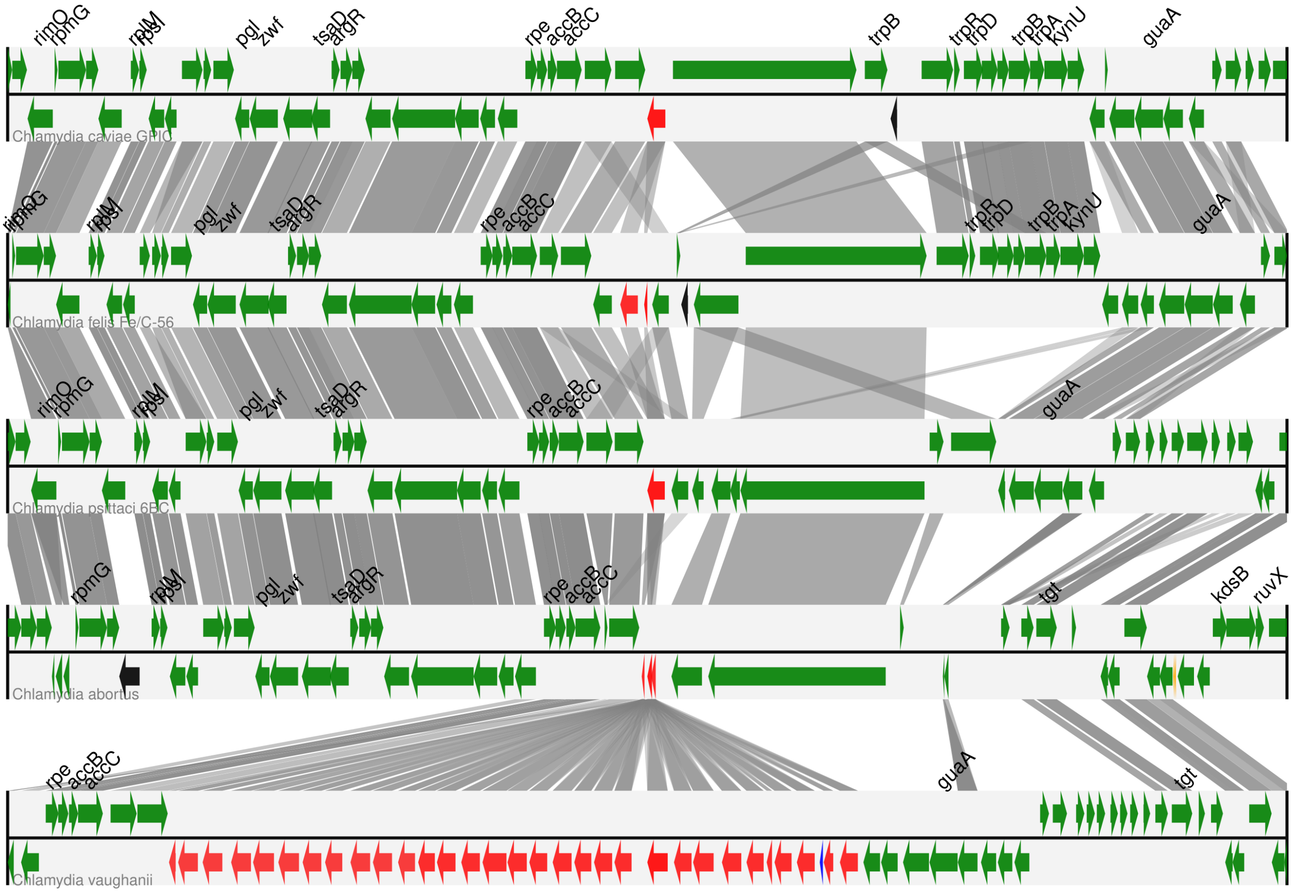

Supp. Fig. 8

Comparison of the genomic regions encoding the 32 repeats of an hypothetical repeats (red arrows). The repeats are located in the plasticity zone. The blue arrow highlights one of the short gene predicted to be unique to *C. vaughanii*. Gray bands link genes from the same orthogroup. Green: protein encoding genes. Black: pseudogenes. Yellow: tRNA encoding gene.

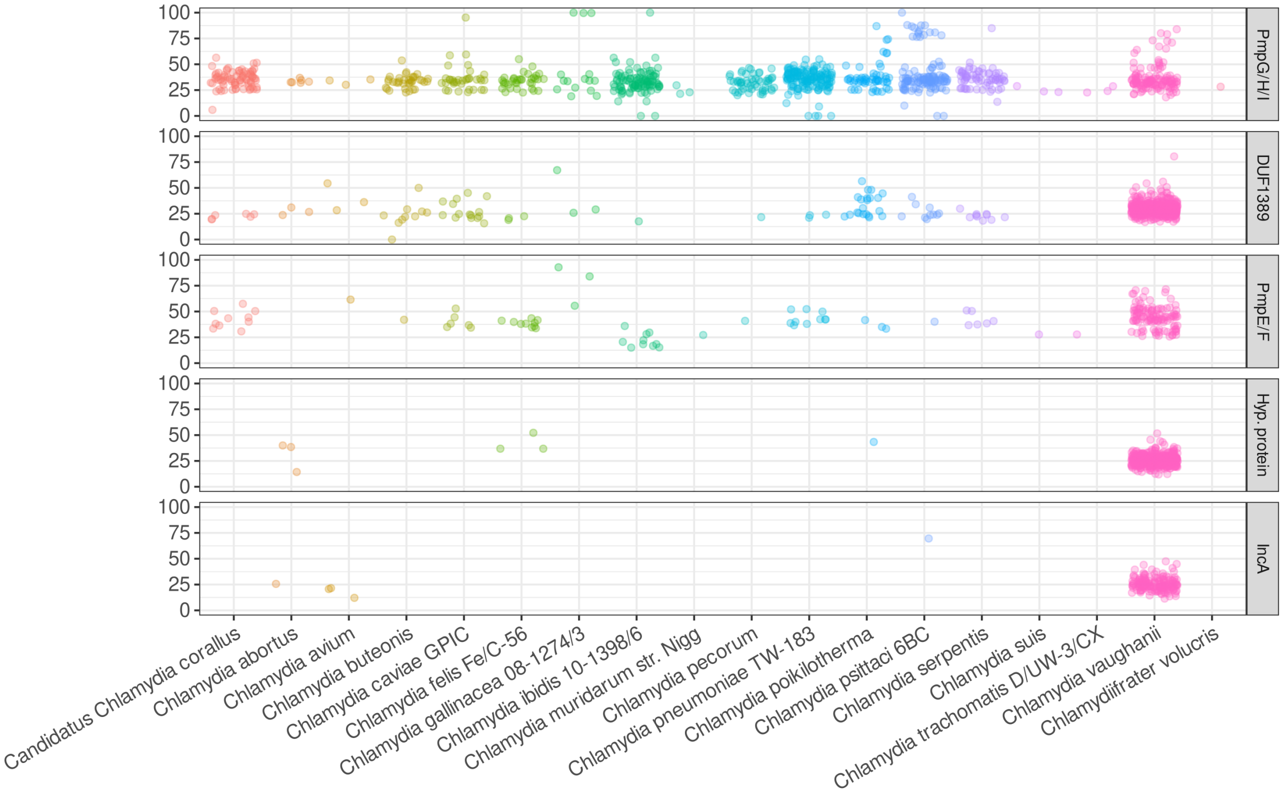

Supp. Fig. 9
Intra-species pairwise identity of the 5 most repeated orthogroups of *C. vaughanii* (as percentage identity). The pairwise identity were computed based on the mafft multiple alignment of the amino-acid sequence of the proteins of each orthogroup. The orthogroups were inferred with Orthofinder. Pmp: polymorphic membrane protein. Inc: inclusion membrane protein. DUF: domain of unknown function.

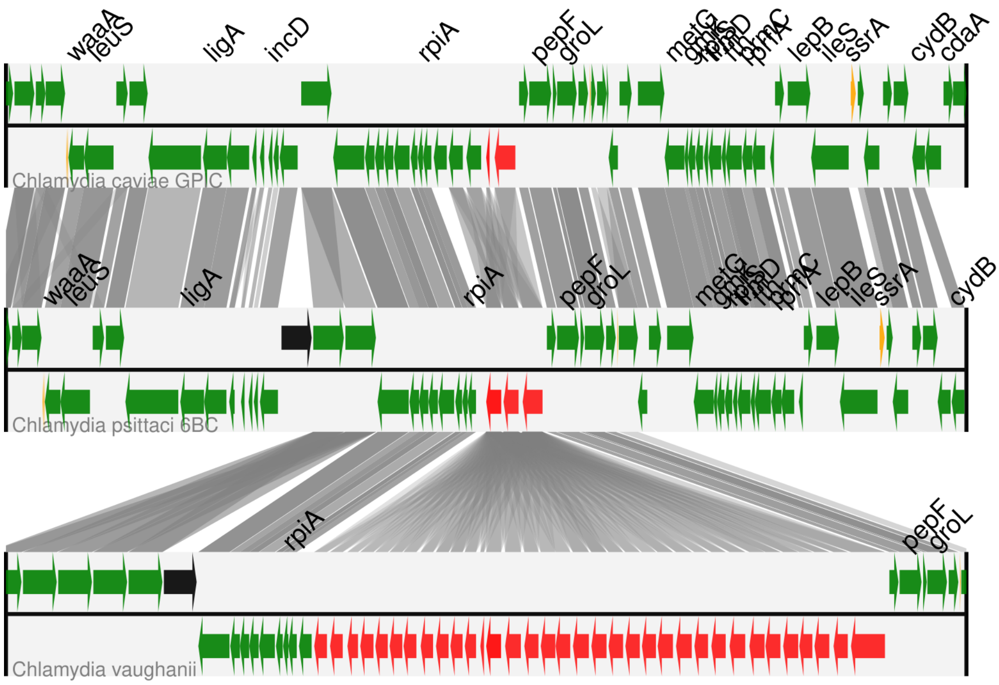

Supp. Fig. 10
Comparison of the genomic regions encoding the repeats of the DUF1389-domain containing proteins (red arrows). Gray bands link genes from the same orthogroup. Green: protein encoding genes. Black: pseudogenes. Yellow: tRNA encoding gene.

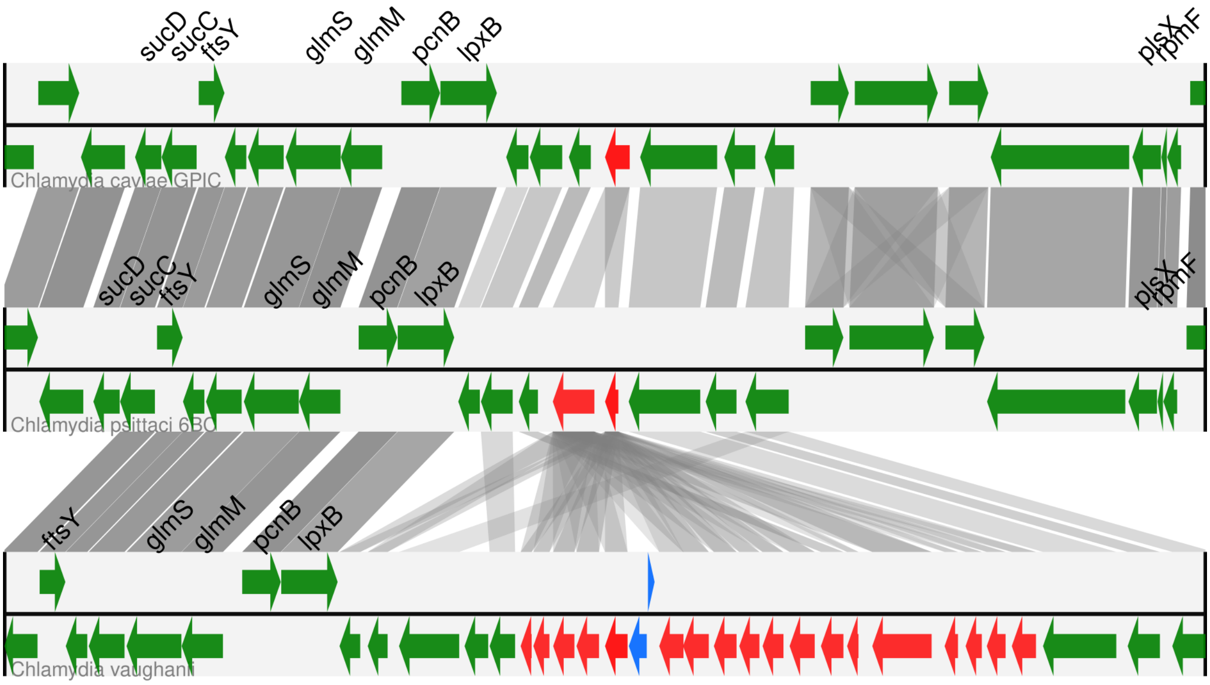

Supp. Fig. 11
Comparison of the genomic regions encoding the repeats of the IncA-family proteins (red arrows). The blue arrow highlights two of the short genes predicted to be unique to *C. vaughanii*. Gray bands link genes from the same orthogroup. Green: protein encoding genes.

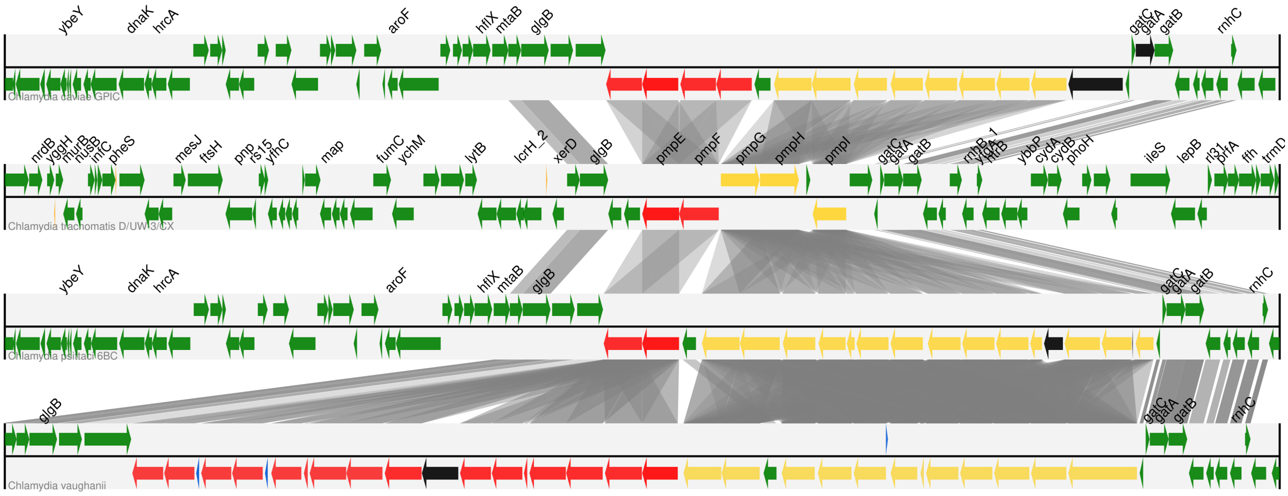

Supp. Fig. 12

Comparison of the genomic regions encoding the repeats of pmpG/H/I (red arrows) and pmpE/F (yellow arrows). The blue arrow highlights three of the short genes predicted to be unique to *C. vaughanii*. Gray bands link genes from the same orthogroup. Green: protein encoding genes. Black: pseudogenes.

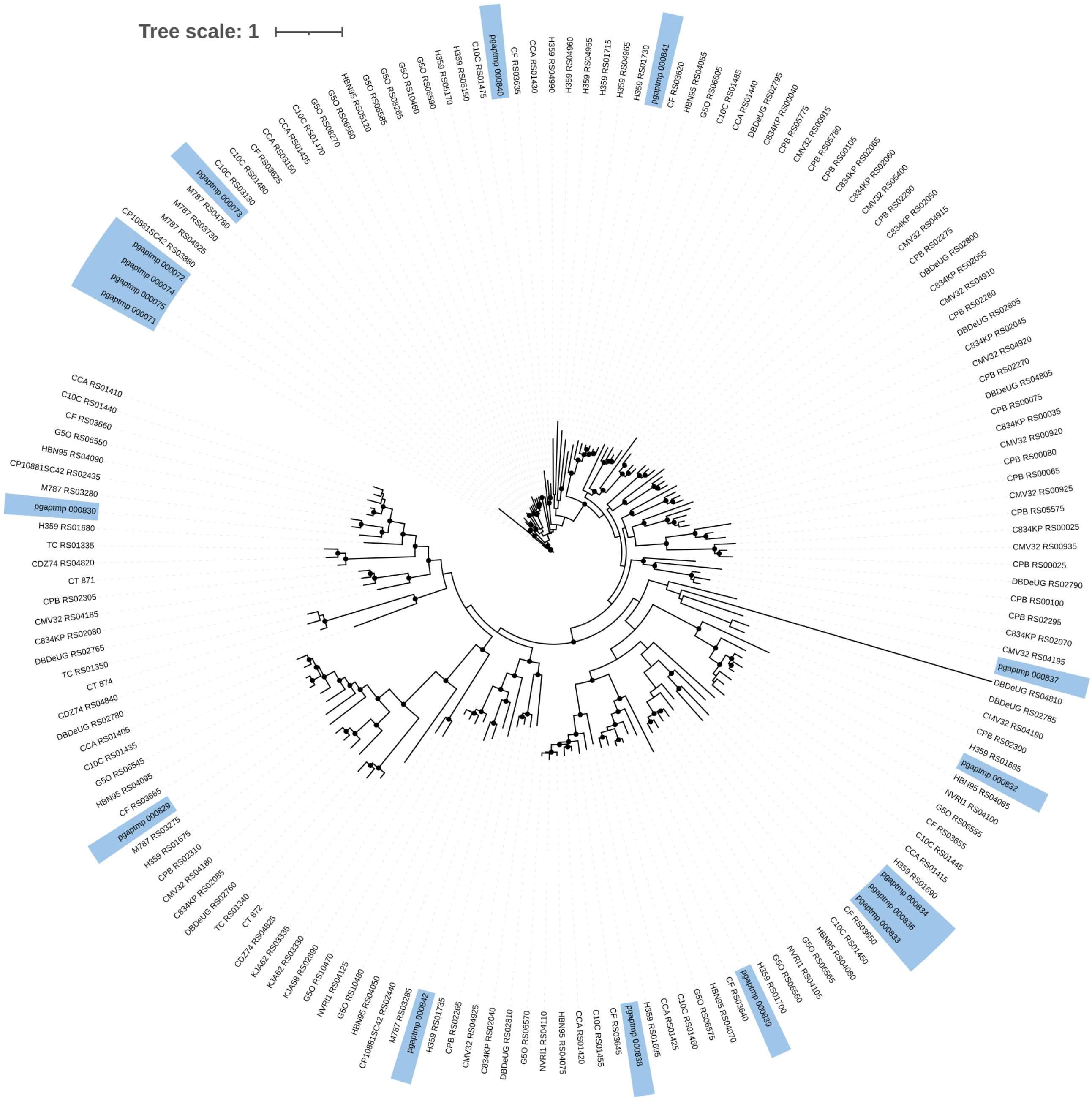

Supp. Fig. 13

Unrooted maximum-likelihood phylogenetic trees of the PmpG/H/I orthogroup. The amino-acid sequences were aligned with mafft. Black dots show nodes with more than 98% support, based on 1000 ultra-fast bootstrap replicates of IQ-tree. *C. vaughanii* branches are highlighted in blue.

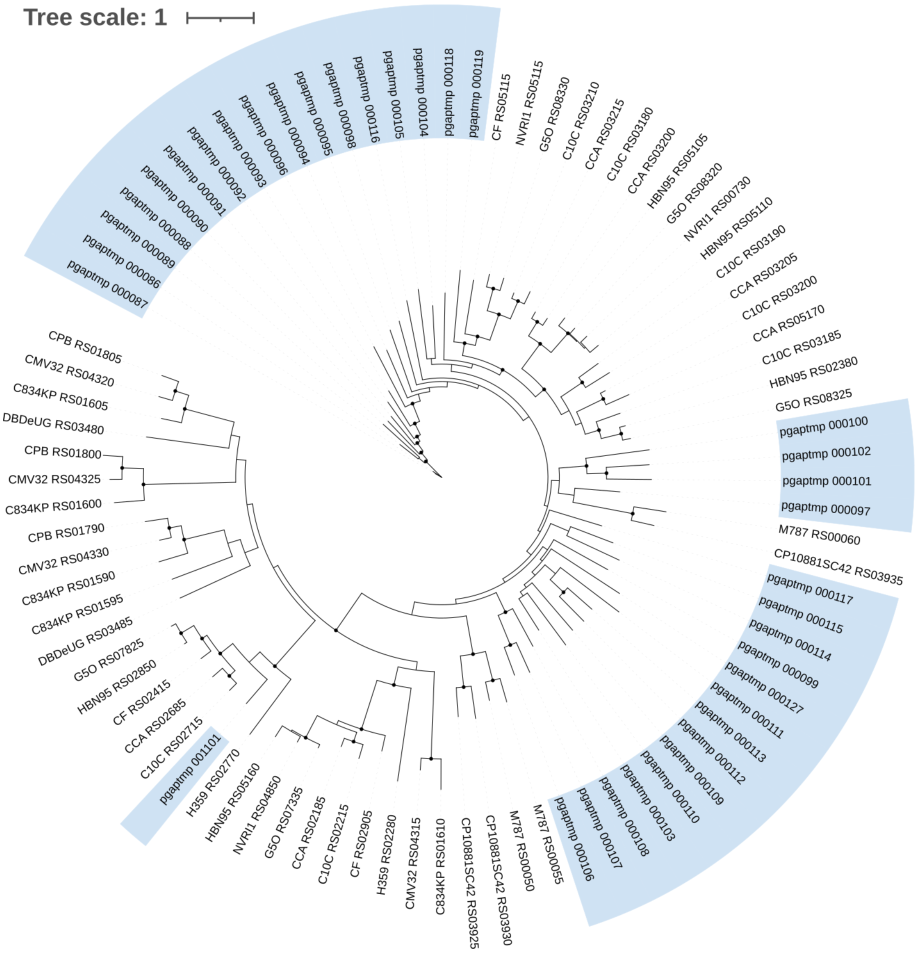

Supp. Fig. 14
Unrooted maximum-likelihood phylogenetic trees of DUF1389-containing protein. The amino-acid sequences were aligned with mafft. Black dots show nodes with more than 98% support, based on 1000 ultra-fast bootstrap replicates of IQ-tree. *C. vaughanii* branches are highlighted in blue.

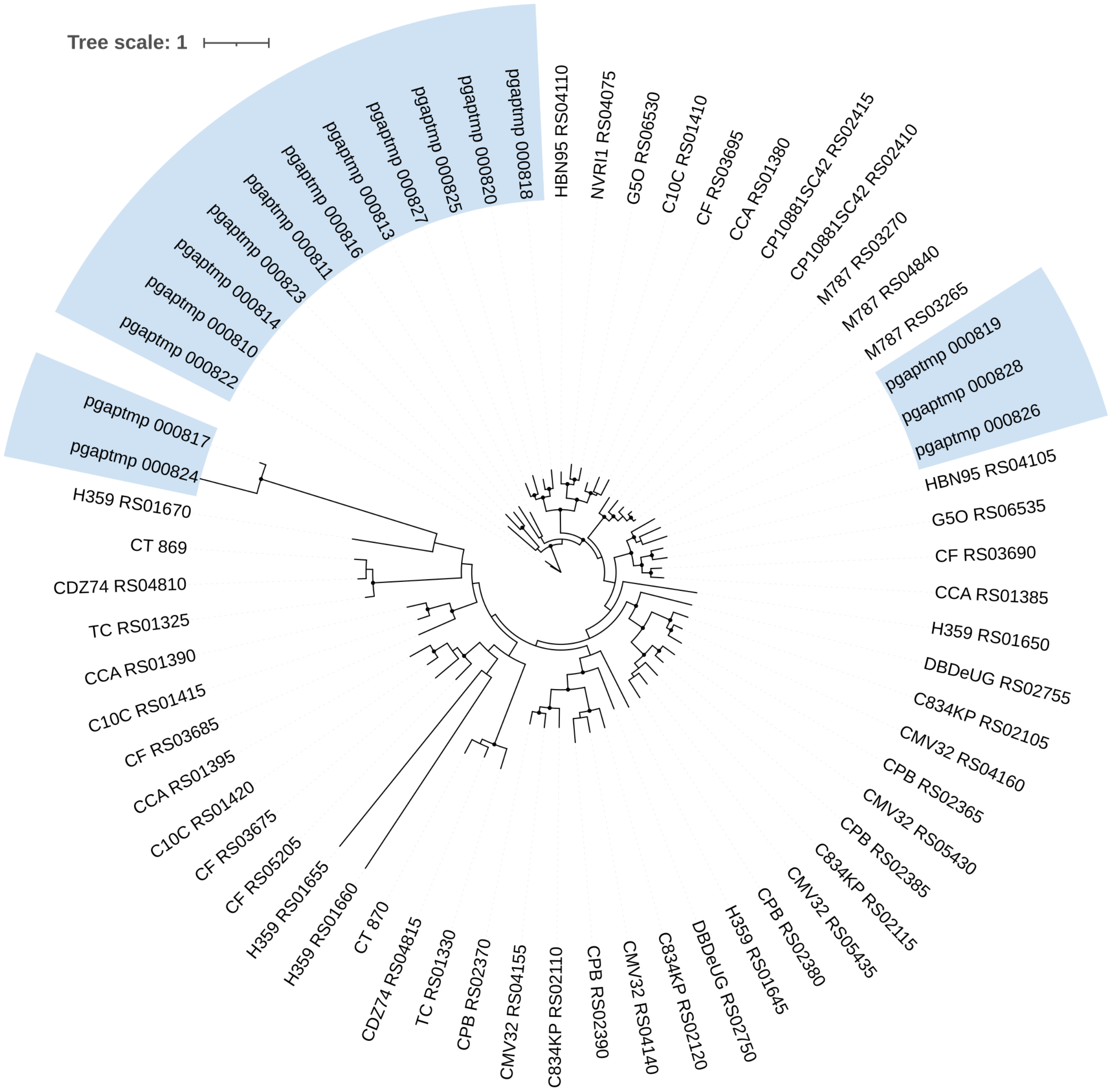

Supp. Fig. 15

Unrooted maximum-likelihood phylogenetic trees of the PmpE/F orthogroup. The amino-acid sequences were aligned with mafft. Black dots show nodes with more than 98% support, based on 1000 ultra-fast bootstrap replicates of IQ-tree. *C. vaughanii* branches are highlighted in blue.

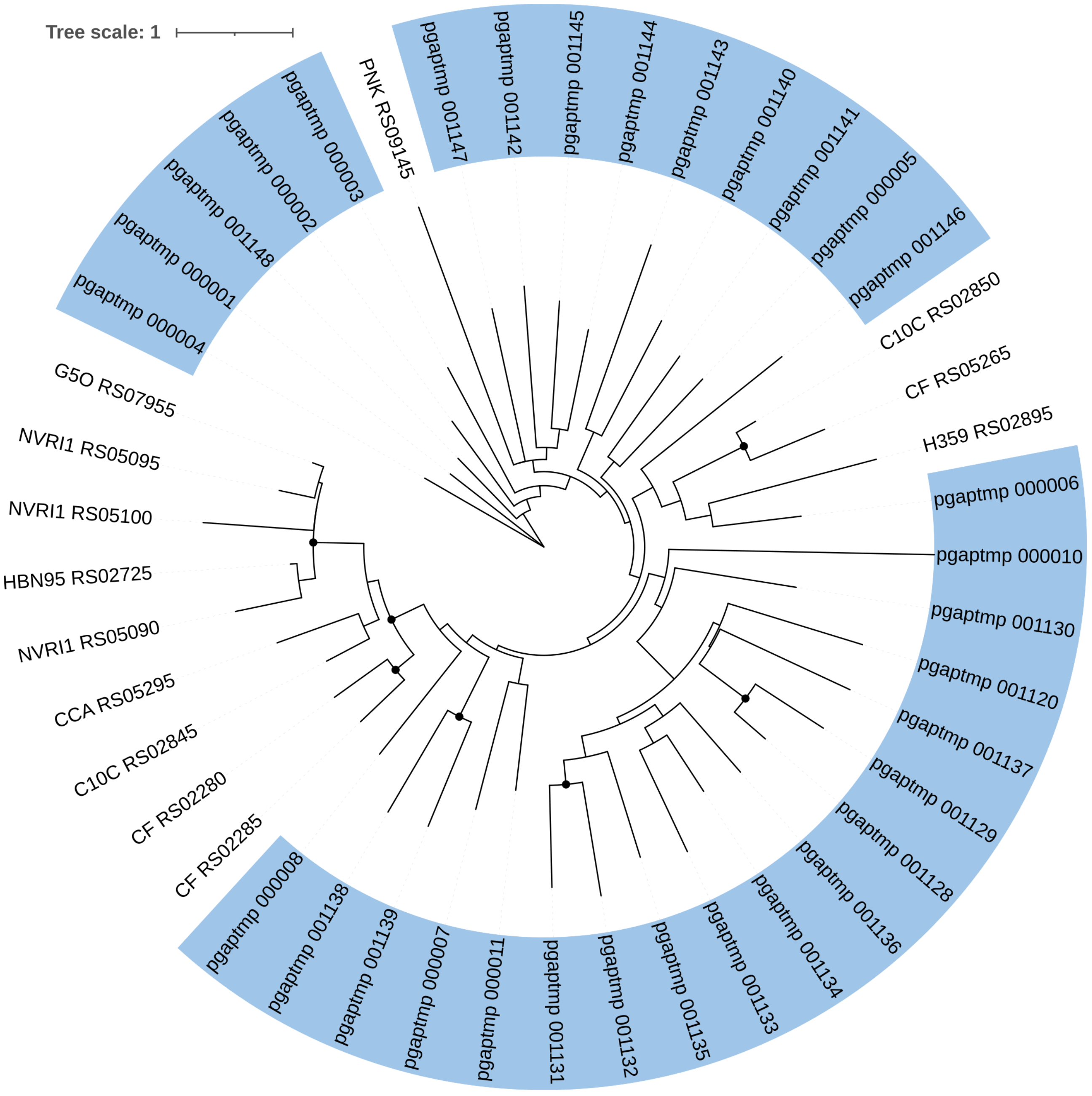

Supp. Fig. 16

Unrooted maximum-likelihood phylogenetic trees of the orthogroup containing 32 repeats of hypothetical proteins in the genome of *C. vaughanii* (Supp. Fig. 6). The amino-acid sequences were aligned with mafft. Black dots show nodes with more than 98% support, based on 1000 ultra-fast bootstrap replicates of IQ-tree. *C. vaughanii* branches are highlighted in blue.

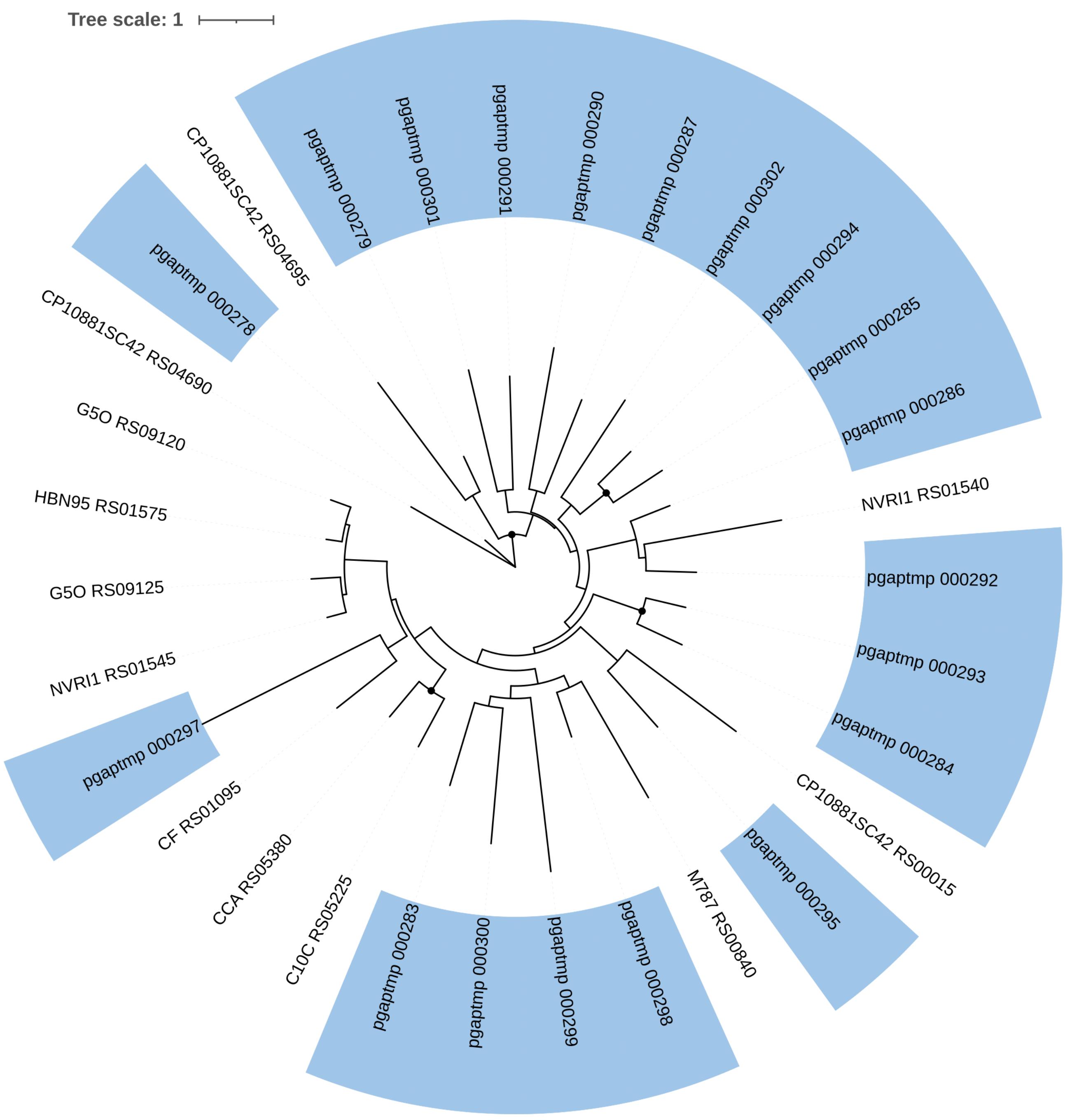

Supp. Fig. 17

Unrooted maximum-likelihood phylogenetic trees of the IncA-homologs orthogroup. The amino-acid sequences were aligned with mafft. Black dots show nodes with more than 98% support, based on 1000 ultra-fast bootstrap replicates of IQ-tree. *C. vaughanii* branches are highlighted in blue.
